# Standardizing social determinants of health data: a proposal for a comprehensive screening tool to address health equity a systematic review

**DOI:** 10.1093/haschl/qxae151

**Published:** 2024-11-14

**Authors:** Sarju Ganatra, Sumanth Khadke, Ashish Kumar, Sadiya Khan, Zulqarnain Javed, Khurram Nasir, Sanjay Rajagopalan, Rishi K Wadhera, Sourbha S Dani, Sadeer Al-Kindi

**Affiliations:** Division of Cardiovascular Medicine, Department of Medicine, Lahey Hospital and Medical Center, Burlington, MA 01805, United States; Division of Cardiovascular Medicine, Department of Medicine, Lahey Hospital and Medical Center, Burlington, MA 01805, United States; Department of Medicine, Cleveland Clinic, Akron General, Akron, OH 44307, United States; Division of Cardiology, Department of Medicine and Preventive Medicine, Northwestern University Feinberg School of Medicine, Chicago, IL 60611, United States; Division of Cardiovascular Prevention and Wellness, Department of Cardiology, Houston Methodist DeBakey Heart and Vascular Center, Houston, TX 77030, United States; Division of Cardiovascular Prevention and Wellness, Department of Cardiology, Houston Methodist DeBakey Heart and Vascular Center, Houston, TX 77030, United States; Harrington Heart and Vascular Institute, University Hospitals and Case Western Reserve School of Medicine, Cleveland, OH 44106, United States; Richard A. and Susan F. Smith Center for Outcomes Research, Beth Israel Deaconess Medical Center, Boston, MA 02215, United States; Division of Cardiovascular Medicine, Department of Medicine, Lahey Hospital and Medical Center, Burlington, MA 01805, United States; Division of Cardiovascular Prevention and Wellness, Department of Cardiology, Houston Methodist DeBakey Heart and Vascular Center, Houston, TX 77030, United States

**Keywords:** social determinants of health, screening tools, health equity, data standardization, community resources

## Abstract

Social determinants of health (SDoH) significantly impacts health outcomes and disparities. While the Centers for Medicare and Medicaid Services has mandated hospitals to collect standardized SDoH data, existing tools lack key elements. This systematic review identified 78 studies and 20 screening tools addressing various SDoH domains. However, most tools were missing several key domains and lacked standardization. We propose a comprehensive tool meeting essential criteria: validated questions, brevity, actionability, cultural appropriateness, workflow integration, and community linkage. Our tool addresses gaps in available tools and incorporates standardized and validated questions to enable patient-centered screening for diverse social and environmental determinants of health. It uniquely includes detailed race/ethnicity data collection, housing characteristics, physical activity assessment, access to healthy food measures, and environmental exposure evaluation. The tool aims to provide actionable data for immediate interventions while informing broader population health strategies and policy initiatives. By offering a holistic assessment of SDoH across multiple domains, our tool enables standardized data collection, risk stratification, and focused initiatives to address health inequities at both individual and population levels. Further research is needed to develop evidence-based pathways for integrating SDoH data into real-world patient care workflows, improve risk prediction algorithms, address health-related social needs, and reduce disparities.

## Introduction

Social determinants of health (SDoH) is increasingly recognized as critical factors influencing health outcomes, healthcare utilization, and health disparities.^1^ These determinants, encompassing economic stability, education, social and community context, health and healthcare, and neighborhood and built environment, impacting an estimated 80% of health outcomes.^2^ The integration of SDoH data into clinical practice and health systems offers transformative potential for improving patient care, reducing health disparities, and optimizing resource allocation.^3^ Systematic collection and analysis of SDoH data can enhance risk prediction models, inform targeted interventions, guide community health initiatives, and facilitate more equitable healthcare delivery.^4^ Moreover, aggregating SDoH data at the population level can inform policy decisions, such as the development of the Social Vulnerability Index (SVI), support community needs assessments, and enable robust evaluations of health equity initiatives.^1^

In a significant policy shift, the Center for Medicare and Medicaid Services (CMS) has mandated that hospitals participating in the Inpatient Quality Reporting program begin submitting 2 new SDoH screening measures, SDoH-1 and SDoH-2, which are mandatory in 2024.^5^ This mandate has created an urgent need for healthcare organizations to implement standardized SDoH screening tools. However, many care providers are struggling to identify the optimal tool for their specific contexts and patient populations.^6,7^ A study by Gottlieb et al.^8^ found that healthcare systems often default to readily available or widely used tools rather than conducting comprehensive evaluations to identify the most effective screening instruments. This approach may lead to the adoption of tools that are not fully aligned with organizational needs, patient populations, or regulatory requirements.

Implementing standardized SDoH screening has the potential to address several barriers present in current clinical settings.^9^ These include limited awareness of patients’ social challenges, missed opportunities for prevention and intervention, a narrow focus on medical treatment and lifestyle counseling, lack of standardization in addressing social needs, inefficient use of healthcare resources, limited integration of social needs into care planning, barriers to patient disclosure of social issues, disconnect between clinical care and community resources, and limited understanding of population-level social needs.^9^ By systematically assessing and documenting SDoH, clinicians can gain a more comprehensive understanding of their patients’ life circumstances, enabling more holistic and patient-centered care.^10^ This approach can lead to earlier identification of social challenges, more targeted interventions, improved care coordination, and more efficient use of healthcare resources.^9^ Moreover, standardization can provide valuable data to inform population health management strategies and strengthen connections between healthcare and community-based services.

Significant heterogeneity in content, format, and implementation of SDoH screening poses challenges for data interoperability, benchmarking, and the development of evidence-based interventions.^10^ Moreover, many existing tools lack comprehensive coverage of all relevant SDoH domains, fail to integrate seamlessly with electronic health records, or do not fully align with CMS requirements.^11^ The absence of a standardized, comprehensive, and widely accepted SDoH screening tool creates a critical gap in the healthcare system's ability to effectively and uniformly identify, address social determinants, and meet regulatory requirements.^7^ Our proposed SDoH tool has the potential to inform more personalized clinical decision-making, facilitate targeted referrals to social services, enable population health management strategies, foster cross-sector collaborations between healthcare and community organizations, and provide standardized data for quality improvement initiatives along with research on SDoH interventions.^12^

To address these challenges and fill the existing gaps, our study aims to accomplish 2 primary objectives, we conduct a systematic review of the existing SDoH screening tool landscape, evaluating the strengths, limitations, and gaps in current instruments. This review provides a comprehensive assessment of available tools, their domains of focus, validation status, and alignment with CMS requirements. Based on the insights gained from this systematic review, we develop a comprehensive, standardized SDoH screening toolkit that not only complies with new CMS requirements but also addresses the limitations identified in existing tools. This toolkit aims to provide healthcare organizations with a robust, evidence-based approach to SDoH screening that can be seamlessly integrated into clinical workflows and electronic health records.

The integration of a comprehensive SDoH screening tool into clinical practice has significant potential to transform patient care and health outcomes. By systematically assessing a wide range of social and environmental factors, clinicians can gain a more holistic understanding of their patients’ lived experiences and the barriers they face in achieving optimal health. This deeper insight enables more personalized and effective care planning, targeted interventions, and improved resource allocation. For example, identifying food insecurity may prompt referrals to local food banks or nutrition assistance programs, while recognizing transportation barriers could lead to the arrangement of medical transportation services or telemedicine options. Moreover, the aggregated data from widespread implementation of such a tool can inform population health strategies, guide community health initiatives, and provide valuable evidence for health policy decisions. By addressing upstream determinants of health, this approach has the potential to reduce health disparities, improve overall population health, and ultimately lead to more efficient use of healthcare resources.

## Methods

### Search strategy and study eligibility

Two authors (S.K. and A.K.) independently identified cross-sectional and longitudinal studies published between 2000 and 2023 that reported on the individual elements of SDoH like housing, income, food insecurity, domestic violence, unemployment, transportation, education, and social isolation by systematically searching MEDLINE/PubMed, CINAHL, EMBASE, and Google Scholar. In addition, the authors screened the reference lists of articles identified and corresponded with study investigators using approaches consistent with the Preferred Reporting Items for Systematic Reviews and Meta-analyses reporting guideline and did not require institutional review board approval as it did not involve human participants. We performed a systematic review of tools that collect SDoH, including an assessment of study quality and quantitative synthesis of outcomes. A third author (S.G.) was involved in case of any discrepancies by mutual discussion and adjudication.

In addition to the systematic literature search, we conducted targeted website searches to identify widely used and validated SDoH screening tools and indices that may not have been captured in the peer-reviewed literature. These searches included websites of government agencies, professional organizations, and healthcare institutions known to be involved in SDoH initiatives. Key search terms included “social determinants of health screening,” “SDoH tools,” “health equity screening,” and “social needs assessment.” Through these supplementary website searches, we identified an additional 20 SDoH screening tools and indices, which are summarized in Table 1 alongside the tools identified from the literature review.

**Table 1.** Various tools to study social determinants of health.

SDoH tools	Description	Advantages	Disadvantages	Components	Use of validated questions	Assess the CMS defined HRSN
County health rankings and roadmaps (CHRR)	CHRR is a program of the University of Wisconsin opulation Health Institute. The rankings are unique in their ability to assess the well-being of nearly every county in all 50 states, and are accompanied by guidance, tools, and resources intended to expedite community learning and action.	The rankings allow comparison of counties across the United States based on various domains of SDoH. The measures used by CHRR are standardized and combined using scientifically informed weights, ensuring a rigorous and evidence-based approach to assessing health outcomes and factors.	Lack of granularity may not accurately reflect the disparities within the county. Does not analyze impact of environmental hazards like air and water pollution, exposure to toxic hazardous sites, lead and coal mines.	☒ Minority status and language ☒ Socioeconomic factors ☒ Financial resource strain □ Household type ☒ Household characteristics ☒ Transportation ☒ Access to food, utilities and healthcare ☒ Social connection ☒ Health behavior ☒ Access to Phone and Internet ☒ Environment burden □ Stress	Yes https://www.countyhealthrankings.org/sites/default/files/media/document/2023%20CHRR%20Technical%20Document.pdf	Yes
Residential segregation index	It is also called as Index of dissimilarity. This index is a measure of the level of segregation or integration between the Black and White populations within a specific geographic area. (ACS)	Able to compare segregation caused by structural, institutional, and individual racism across the country. It helps in analyzing structural inequalities, socioeconomic mobility, and healthcare access.	It is essential to consider additional qualitative and quantitative data to analyze factors contributing to residential segregation and its health implications. All that apply to CHRR	☒ Minority Status and language □ Socioeconomic factors □ Financial resource strain □ Household type □ Household characteristics □ Transportation □ Access to food, utilities and healthcare □ Social connection □ Health behavior □ Access to Phone and Internet □ Environment burden	Yes https://www.countyhealthrankings.org/sites/default/files/media/document/2023%20CHRR%20Technical%20Document.pdf	Partial
Food environment index	This index measures the ability to access healthy foods accounting for the socioeconomic status and distance needed to travel to supermarkets. It equally weight 2 indicators a) Limited access to healthy foods (low income and remoteness to groceries) b) Food insecurity	This tool helps us compare the counties based on either limited access to healthy food and food insecurity indicators.	The food insecurity models include state-level effects that may overestimate differences in border counties. Does not account for environmental hazards like exposure to toxic sites, air and water pollution. Tracking individual county improvement is impossible due to the scaled nature of the measure. All that apply to CHRR.	□ Minority Status and language □ Socioeconomic factors ☒ Financial resource strain □ Household type □ Household characteristics □ Transportation ☒ Access to food, utilities and healthcare □ Social connection □ Health behavior □ Access to Phone and Internet □ Environment burden □ Stress	Yes https://www.countyhealthrankings.org/sites/default/files/media/document/2023%20CHRR%20Technical%20Document.pdf	Partial
The California Healthy places index (HPI)	The HPI is a project of the Public Health Alliance of Southern California, a coalition of the executive leadership of 10 local health departments in Southern California, representing more than 60% of the state's population. HPI is a tool to explore the community conditions that impact life expectancy. It combines 25 community characteristics, like access to healthcare, housing, education, and more, into a single indexed HPI score.	Each HPI indicator is linked to our Policy Action Guide, which highlights equitable solutions to improving community health. It allows analysis at multiple levels, including census tracts, cities, and counties. Compares data across geographies and time periods. Can visualize historically redlined neighborhoods. The HPI allows users to customize the index based on their specific needs by selecting the indicators and weights that align with their research or policy objectives. Includes Environment aspects like air and water quality. As most of the individual indicators in the HPI are sourced from national datasets, a national HPI is feasible. The index can study SDoH in time series to help assess effect of interventions on equity.	The HPI is designed specifically for California, limiting its applicability to other regions or states. The HPI relies on various data sources, and the quality, availability, and timeliness of these sources may vary. In some cases, data may be outdated or incomplete, which can affect the accuracy and reliability of the index. Does not measure environmental burden as comprehensively as compared with EJI. Findings are sensitive to mass evacuation due to wildfires and climate emergencies, gentrification, community succession, and displacement	□ Minority Status and language ☒ Socioeconomic factors ☒ Financial resource strain □ Household type □ Household characteristics ☒ Transportation □ Access to food, utilities, and healthcare □ Social connection □ Health behavior □ Access to Phone and Internet ☒ Environment burden □ Stress	Yes https://empowerla.org/wp-content/uploads/2020/08/Sadler_HPI-Presentation-to-LA-NB-Commn-8-17-2020.pdf	Partial
Baseline Resilience Indicators for Communities (BRIC)	The BRIC helps study the differences in community resilience among counties within the state and the nation through a comparative community resilience score. BRIC considers 6 broad categories of community disaster resilience: social, economic, community capital, institutional, infrastructural, and environmental at the county level.	It Compare the community resilience among counties and census tracts within the state and within the nation. BRIC data can compare or track community resilience changes over time.	BRIC utilizes uniform formulas and variables across the coverage area and does not consider community-specific variables. It does not include the prevalence or existence of hazards in the vulnerability equation except social factors. This index should be used in conjunction with hazard information. Provides a single snapshot of a community. Multiple events and duration of events not included in the index may further impact a community's vulnerability at a given time.	☒ Minority Status and language ☒ Socioeconomic factors □ Financial resource strain □ Household type □ Household characteristics ☒ Transportation ☒ Access to food, utilities and healthcare □ Social connection □ Health behavior ☒ Access to Phone and Internet □ Environment burden □ Stress	Yes https://www.mdpi.com/2073-4441/12/5/1401	Partial
Community resilience estimates (CRE)	(CRE) provide an easily understood metric for how at-risk every neighborhood in the United States is to the impacts of disasters, including COVID-19. CRE is calculated using ACS.	It uses restricted microdata housed at the U.S. Census Bureau, that allows CRE to cumulatively flag persons living with a social vulnerability risk factor. Uses small area estimation allows CRE to avoid statistical noise. CRE is only social vulnerability and resilience data product to use 1-year ACS data while also having complete tract coverage for the U.S.	Constant set of indicators are being used to calculate the CRE. Use of microdata and small area estimation, one cannot know what the indicators that are impacting CRE of a tract or county from the model.	☒ Minority Status and language (Language) ☒ Socioeconomic factors ☒ Financial resource strain ☒ Household type(crowding) ☒ Household characteristics (Disability) ☒ Transportation □ Access to food, utilities and healthcare □ Social connection □ Health behavior ☒ Access to Phone and Internet □ Environment burden □ Stress	Yes https://www2.census.gov/programs-surveys/acs/methodology/design_and_methodology/2022/acs_design_methodology_report_2022.pdf	Partial
Child Opportunity Index (COI)	First single metric of contemporary child neighborhood opportunity. An index of neighborhood resources and conditions that help children develop healthily. It combines data from 29 neighborhood-level indicators across 3 opportunity SDoH domains: a) Education, b)Health, and environment, c) Socioeconomic aspects.	Measures the racial and ethnic opportunity gaps among children. Longitudinal data is available for comparison. Complete national coverage: provides data for nearly all U.S. neighborhoods (about 72 000 census tracts) It uniquely assesses access to green space and healthy food, and accounts for air pollution, hazardous waste dump sites, Industrial pollutants in air, water or soil, ozone concentration and extreme heat	It lacks relevant for children like, neighborhood-level prevalence of violent crime, neighborhood social capital and collective efficacy, transportation costs and provider-side measures of access to health care (eg, density of primary care physicians or pediatricians)	☒ Minority Status and language ☒ Socioeconomic factors ☒ Financial resource strain □ Household type □ Household characteristics ☒ Transportation ☒ Access to food, utilities and healthcare ☒ Social connection □ Health behavior □ Access to Phone and Internet ☒ Environment burden □ Stress	Yes https://www.diversitydatakids.org/sites/default/files/2020-02/ddk_coi2.0_technical_documentation_20200212.pdf	Partial
Distressed community index (DCI)	This tool measures the comparative economic well-being of U.S. communities and helps illuminate ground-level disparities across the country. The DCI divides zip codes into quintiles of well-being: prosperous, comfortable, mid-tier, at risk, and distressed.	The DCI combines 7 complementary economic indicators into a single score and sorts of communities into 5 even tiers, or quintiles, of well-being: prosperous, comfortable, mid-tier, at risk, and distressed.	The tool cannot screen for the remaining social needs like Health behaviors, outcomes, minority segregation, food insecurity and environmental burden.	□ Minority Status and language ☒ Socioeconomic factors ☒ Financial resource strain □ Household type □ Household characteristics □ Transportation □ Access to food, utilities and healthcare □ Social connection □ Health behavior □ Access to Phone and Internet □ Environment burden □ Stress	Yes https://www2.census.gov/programs-surveys/acs/methodology/design_and_methodology/2022/acs_design_methodology_report_2022.pdf	Partial
National walkability index (NWI)-2021	The NWI is based on measures of the built environment that affect the probability of people choosing to walk as the mode of transportation.	It analyzes and compares Community’s walkability It can be used in rural areas to identify walkable areas. The dataset covers every block group in the nation, providing a basis for comparing walkability from community to community.	Does not measure other socioeconomic factors like Education; Employment; Income; Family support; Community safety	□ Minority Status and language ☒ Socioeconomic factors □ Financial resource strain □ Household type □ Household characteristics □ Transportation □ Access to food, utilities and healthcare □ Social connection □ Health behavior □ Access to Phone and Internet □ Environment burden □ Stress	Statistically derived index	Partial
Social deprivation index (SDI)	SDI is a composite measure of area-level deprivation based on 7 demographic characteristics collected in the ACS and used to quantify the socioeconomic variation in health outcomes. It helps quantify levels of disadvantage across small areas, evaluate their associations with health outcomes, and address health inequities. The SDI measures were developed by Butler et al. (2012) using 2005–2009 ACS 5-year estimates.	Avails measures at 4 geographic levels: county, census tract, ZCTA (Zip Code Tabulation Areas), and PCSA** (Primary Care Service Areas) which helps to study social deprivation at various levels of granularity. SDI permits the assessment of the individual SDoH components to understand the nuanced population effects or socioeconomic variability in health outcomes of social determinants of health.	Calculation of SDI excludes percent black and percent in high-needs age groups. Calculation of SDI may be prone to loss of information due to conversion of measures into centiles. May pose challenges in interpretation due to the use of centiles with factor analysis.	□ Minority Status and language ☒ Socioeconomic factors □ Financial resource strain □ Household type ☒ Household characteristics ☒ Transportation □ Access to food, utilities and healthcare □ Social connection □ Health behavior □ Access to Phone and Internet □ Environment burden □ Stress	Yes https://www2.census.gov/programs-surveys/acs/methodology/design_and_methodology/2022/acs_design_methodology_report_2022.pdf	Partial
Whole person health score	A patient focused tool to measure SDoH. A questionnaire including 26 questions categorized into 6 domains like Physical Health, Emotional Health, Resource Utilization, Socioeconomics, Ownership, Nutrition and Lifestyle.	Integrates and directly measures factors like self-management, self-efficiency, mental health, and social support. Tracks the progress in terms of SDoH at individual level. WPHS may help to recognize and financially quantify the contributions of nonbillable health care providers and stakeholders. The visual and scoring elements of the WPHS nudges health care teams to prioritize nontraditional upstream patient needs, including emotional health, ownership, and SDoHs, and encourages engaging patients in their own care. The nature of WPHS as a quantified metric allows for tracking SDoH changes over time, simplifies the interpretation of a patient's SDoH status, and allows for the comparison and ranking of possible areas needing interventions	It grades patients from A(best) to Z (worst) scores, which are categorized into 3 categories based on severity. Ranks A–F are coded as green and indicates low need; G–O is yellow which indicates moderate need; and P–Z is red which indicates high need, a trigger that the domain is impacting the patient's health and requires immediate attention. It does not account for the impacts of environmental burden and social isolation. Integration into EMR and lack of standardized interpretation may limit its applicability.	□ Minority Status and language ☒ Socioeconomic factors ☒ Financial resource strain □ Household type □ Household characteristics □ Transportation ☒ Access to food, utilities, and healthcare □ Social connection ☒ Health behavior (lifestyle) □ Access to Phone and Internet □ Environment burden □ Stress	Yes https://www.careinnovations.org/wp-content/uploads/Geoff-Leung-MD-RUHS-Race-Ethnicity-and-WPHS-08.19.2020-CCI.pdf	Partial
PRAPARE tool	A standardized national patient risk assessment protocol for evaluating and addressing SDoH. This protocol incorporates screening for various domains of SDoH, including personal characteristics, family and home environment, financial resources, and social and emotional well-being. PRAPARE emphasizes measures that are actionable.	Measures the emotional health domain by integrating social integration and depression screening. The tool collects patient level data and can be used to individualize treatments accordingly. PRAPARE uses a risk stratification model with fully automated workflow. The risk score includes SDoH component, clinical component, mental health and substance abuse score, and utilization component score and stratifies patients into emergent/urgent need, high, average, and low need.	The tool does not screen for health influencing behaviors like Tobacco use; Diet and exercise; Alcohol and drug use; Sexual activity (STI and Teen births);	☒ Minority Status and language ☒ Socioeconomic factors ☒ Financial resource strain □ Household type □ Household characteristics ☒ Transportation ☒ Access to food, utilities and healthcare ☒ Social connection □ Health behavior □ Access to Phone and Internet □ Environment burden ☒ Stress	Yes https://aapcho.org/wp-content/uploads/2021/02/prapare_validation-fact-sheet-2019-9-26.pdf	Partial
Area deprivation index (ADI)	ADI enables ranking neighborhoods based on socioeconomic disadvantage within a specific region of interest, such as a state or the entire nation. It encompasses income, education, employment, and housing quality factors, representing the key theoretical domains associated with socioeconomic disadvantage.	Index is refined, adapted, and validated to the Census Block Group neighborhood level. Can be used for resource allocation in the setting of pandemic or epidemic.	Inability to interpret the effect of race and ethnicity as they are not measured. Does not account for environmental burden and is not inclusive of all social disadvantages. Selective focus on economic deprivation. Results are subject to the accuracy and errors contained within the ACS data release	□ Minority Status and language ☒ Socioeconomic factors □ Financial resource strain □ Household type □ Household characteristics □ Transportation □ Access to food, utilities and healthcare □ Social connection □ Health behavior □ Access to Phone and Internet □ Environment burden □ Stress	Yes (https://www.ncbi.nlm.nih.gov/pmc/articles/PMC1447923/)	Partial
CDC SVI	SVI utilizes data from the U.S. Census to evaluate 16 social factors, such as poverty, limited access to transportation, and overcrowded housing. It categorizes them into 4 interconnected themes: •Socioeconomic status, •Household characteristics, •Race and ethnicity, and •Housing type and transportation.	It helps in disaster preparation and prioritizes the communities that need additional interventions. Helps prioritize policy making by identifying census tracts or counties at risk for hazardous events, such as disease outbreaks and natural disasters. It screens for social risks among the communities.	SVI does not account for the environmental burden and not inclusive of all social disadvantages. It does not assess health behaviors, and health outcomes. It does not screen for food insecurity and social isolation	☒ Minority Status and language ☒ Socioeconomic factors □ Financial resource strain ☒ Household type ☒ Household characteristics ☒ Transportation □ Access to food, utilities, and healthcare □ Social connection □ Health behavior □ Access to Phone and Internet □ Environment burden □ Stress	Yes https://www2.census.gov/programs-surveys/acs/methodology/design_and_methodology/2022/acs_design_methodology_report_2022.pdf	Partial
CDC EJI	The EJI is a screening instrument designed to assess the combined impacts of social vulnerability, health and environmental burden on national human health and health equity. EJI also scores communities on each of the 3 modules in the tool (social vulnerability, environmental burden, health vulnerability) and allows more detailed analysis within these modules	The tool measures environmental hazard but also compounds the effects of social factors such as poverty, race, and ethnicity, along with preexisting health conditions may increase these impacts. It analyzes the unique, local factors driving cumulative impacts on health to inform policy and decision-making by public health officials. Measures Health vulnerability by assessing prevalence of Asthma, cancer, Hypertension, Diabetes and Poor mental health. Measures cumulative socioeconomic vulnerability, Environmental burden and Health Vulnerability to obtain a single score.	Not a definitive tool to label environmental justice communities Not a holistic representation of current or future social, environmental, or health characteristics. Ranks each census tract (Each census tract represents a county and is home to an average of 4000 people), hence cannot be individualized to patients. EJI does not screen for food insecurity, social isolation, and health behavior.	☒ Minority Status and language ☒ Socioeconomic factors □ Financial resource strain ☒ Household type ☒ Household characteristics ☒ Transportation □ Access to food, utilities, and healthcare □ Social connection □ Health behavior □ Access to Phone and Internet ☒ Environment burden □ Stress	Yes https://www2.census.gov/programs-surveys/acs/methodology/design_and_methodology/2022/acs_design_methodology_report_2022.pdf	Partial
The Accountable Health Communities HRSNs Screening tool	Drawing from a team of nationwide experts and an examination of current screening tools, CMS crafted a concise 10-item questionnaire to pinpoint patient requirements in 5 vital domains—housing instability, food insecurity, transportation challenges, utility assistance needs, and interpersonal safety. This screening instrument can be employed across varied ages, backgrounds, and environments, making it an efficient addition to bustling clinical workflows.	The tool screens the ability to have stable housing, family and community support, physical activity and substance abuse, mental health, and disability. The tool assesses food insecurity and social isolation.	Designed for Medicare and Medicaid population only. Does not assess medical insurance and healthcare accessibility. Does not account for environmental changes that could influence the effects of other SDoH.	□ Minority Status and language ☒ Socioeconomic factors ☒ Financial resource strain □ Household type ☒ Household characteristics (Disability) ☒ Transportation ☒ Access to food, utilities, and healthcare ☒ Social connection ☒ Health behavior □ Access to Phone and Internet □ Environment burden □ Stress	Yes https://nam.edu/wp-content/uploads/2017/05/Standardized-Screening-for-Health-Related-Social-Needs-in-Clinical-Settings.pdf	Yes
The Health Leads screening toolkit	The social needs screening toolkit has been developed by Health leads, Institute of Medicine and centers for Medicare and Medicaid Services.	− The tool measures homelessness, unsafe or unhealthy housing conditions, utility needs like access to phone, financial resource strain (Inability to afford essential needs, financial literacy, medication use due to cost, transportation challenges, exposure to violence, and Sociodemographic information. Depending on the goals of the initiative, optional social determinants can be included in the health screening tool like, Childcare, Education, Employment, Health behaviors, Social Isolation and support system with behavioral and mental health.	The toolkit does not account for environmental hazards like exposure to PM2.5, water pollution, potentially toxic hazardous sites, natural calamities, that could influence the effects of other SDoH on patient’s health and well-being.	□ Minority Status and language ☒ Socioeconomic factors ☒ Financial resource strain □ Household type □ Household characteristics ☒ Transportation ☒ Access to food, utilities, and healthcare (Food) □ Social connection □ Health behavior ☒ Access to Phone and Internet □ Environment burden ☒ Stress	Yes https://nursing.utexas.edu/sites/default/files/AMEN_Health_Leads_Social_Needs_Screening_Toolkit.pdf	Yes
BMC Thrive	This tool has been developed by clinician researchers based on the E.H.R model that facilitates an automatic print out of referral information for resources based at the hospital and in the community when the patient asks for help with a need they have identified in the screener.	It screens for difficult housing situation, food insecurity, economic instability causing patients to have less access to healthcare and worry about food security.	It does not screen for health behaviors, health outcomes, minority status. It does not screen for lack of adequate health insurance coverage and environmental burden on patients.	□ Minority Status and language ☒ Socioeconomic factors ☒ Financial resource strain □ Household type □ Household characteristics □ Transportation ☒ Access to food, utilities, and healthcare (food) □ Social connection □ Health behavior □ Access to Phone and Internet □ Environment burden □ Stress	Yes https://par.nsf.gov/servlets/purl/10163067	Partial
American Academy of family physicians	These tools can be used by family physicians and their practice teams to screen their patients for social determinants of health, identify community-based resources to help them, and work with patients to develop an action plan that encompasses social needs to help them overcome health risks and improve outcomes.	The social needs screening tool screens for 5 core HRSNs, which include housing, food insecurity, transportation, utilities, and personal safety, using validated screening questions, as well as the additional needs of employment, education, childcare, and financial strain.	Screens patients selectively based on a scoring system unique to the tool. The tool does not assess healthcare access, racial and ethnic status, and environmental burden that could influence the effects of other SDoH.	□ Minority Status and language ☒ Socioeconomic factors ☒ Financial resource strain □ Household type □ Household characteristics ☒ Transportation ☒ Access to food, utilities, and healthcare □ Social connection □ Health behavior □ Access to Phone and Internet □ Environment burden ☒ Stress	Yes* https://www.aafp.org/dam/AAFP/documents/patient_care/everyone_project/sdoh-guide.pdf	Partial
EPIC SDoH Tool	The EPIC SDoH tools are series of questionnaires integrated in the EPIC E.H.R system that document the patient level SDoH data in a systematic way.	Documents clinically validated SDoH assessments for several domains, including Food Insecurity and EPIC connects patients with community resources like food banks to help address the SDoH needs. Allows patients to document their own SDoH. Filtering the SDoH assessments that appear for each patient based on the patient's unique situation. Overriding the settings for an Epic-released domain, allows one to use superior tools to collect data pertaining to a specific SDoH domain.	The system tracks data when a patient's SDoH status has changed but not how often it was assessed. Only data for certain Epic-released SDoH domains are exchanged via Care Everywhere Additional custom workflows are required to enquire if the patient needs SDoH related help. Referrals to resources are not automatically generated. HCO’s need to implement coordinated workflow with community resources. HCO’s may need to work with a third-party vendor to provide a directory of community resources and a means of communicating with those resources.	□ Minority Status and language ☒ Socioeconomic factors ☒ Financial resource strain □ Household type □ Household characteristics ☒ Transportation ☒ Access to food, utilities, and healthcare ☒ Social connection ☒ Health behavior □ Access to Phone and Internet □ Environment burden ☒ Stress	Yes*	Yes

**These tools have both validated and nonvalidated questions.**

**Socioeconomic factors:** Education; Employment; Income; Health insurance, Family support; Community safety. **Health Behaviors:** Tobacco use; Diet and exercise; Alcohol and drug use; Sexual activity (STI and Teen births); **Financial resource strain:** composed of cognitive, emotional, and behavioral responses to financial hardship where an individual cannot meet financial obligations. It is more than just income and encompasses other core needs, such as housing instability and food insecurity. Individuals experiencing financial strain may forgo medical care and prescriptions in order to meet their essential needs, such as housing and food, and may make more affordable, but less healthy food choices. **Housing instability:** encompasses a number of challenges, such as having trouble paying rent, overcrowding, moving frequently, or spending the bulk of household income on housing. **Housing characteristics:** Aged 65 and Older, Aged 17 and Younger, Civilian with disability, or Single-Parent Households. **Housing type:** group homes or Mobile homes **Environmental Burden:** Air pollution due to Ozone, PM 2.5, Diesel particulate matter, Air toxics cancer risk, and exposure to potentially hazardous and toxic sites (Coal, Lead mines, treatment storage and disposal facilities).

Abbreviation: CMS, Center for Medicare and Medicaid Services; HRSN, Health related social needs; SDoH, social determinants of health.

### Inclusion and exclusion criteria

**Figure 1.**
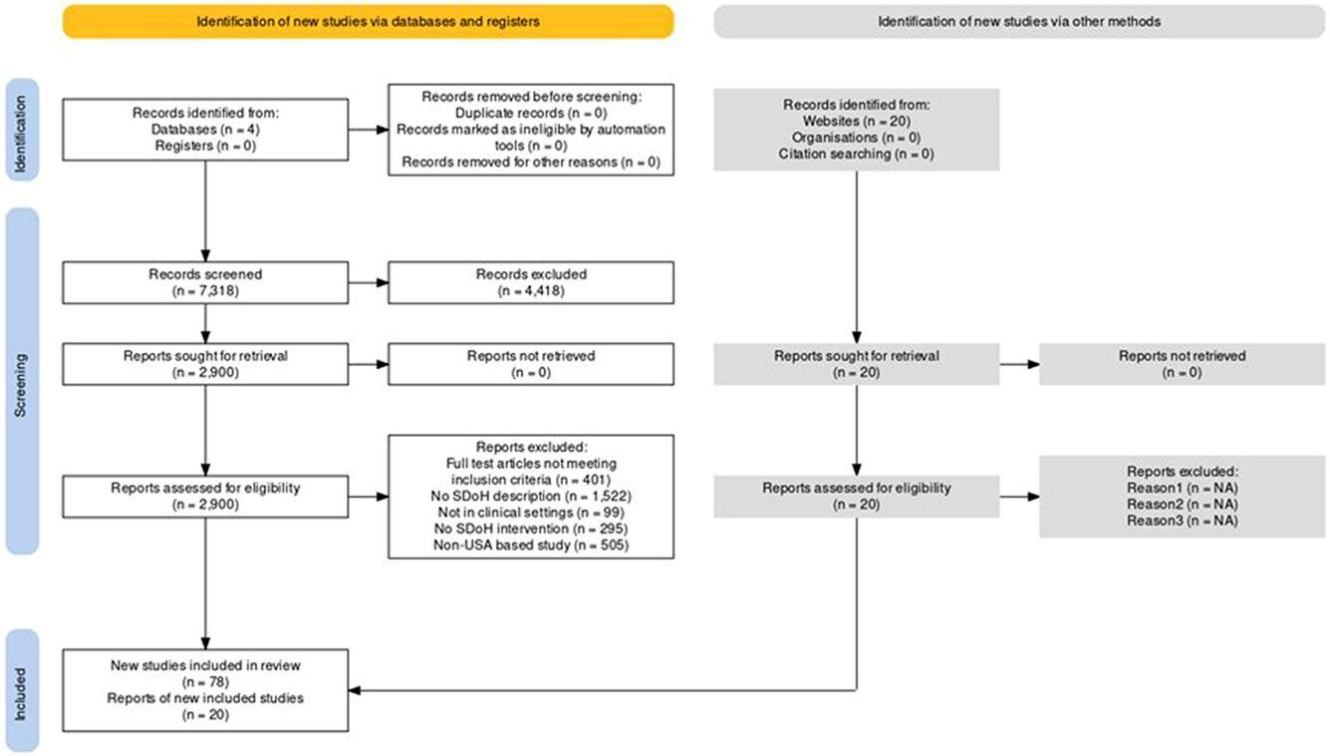
Study identification and review of SDoH screening tools and indices.

To meet inclusion criteria, studies were required to be based in the United States, published between January 2000 and December 2023, and address at least 1 social determinant of health (housing, employment, food, education, interpersonal violence, social isolation, legal services, childcare, or transportation) be integrated within clinical healthcare delivery, and include an evaluation of SDoH component. Programs were considered clinically integrated if patients’ social or economic needs were identified within the clinical setting or through the healthcare system (eg, via demographics or claims data). Following clinical identification of needs, patients may have received social services directly through an internal healthcare program or been linked to external community organizations. As this review focused on the documentation of interventions embedded in clinical settings, the integration criterion aimed to identify healthcare-based rather than community-level interventions except during the pandemic. We also searched other databases and websites which developed validated SDoH tools and included 20 most widely used health-related social needs (HRSNs) screening tools as described in Figure 1 and Table 1.

Studies were excluded if they examined medical care or health behavior interventions only and if they recorded SDoH measures without screening tool implementation from retrospective databases, position statements comparing 2 interventions based on prescreened population. Additionally, although lack of healthcare access exacerbates disparities, references solely describing access interventions like mobile health, telemedicine, or insurance enrollment were excluded given the focus on screening distinct social determinants. We also excluded studies not conducted in the United States and not in English.

For the purposes of this review, we defined “incorporated into clinic workflows for SDoH screening” as any tool or process that was actively used within clinical settings to assess patients’ SDoH. This ranged from well-integrated electronic health record (EHR)-based tools to paper forms manually administered by clinic staff. We included tools that were administered during patient visits, as part of intake processes, or through patient portals, as long as they were directly connected to clinical care. We excluded studies that collected SDoH data in nonclinical settings like population-based screening without clinical follow-up, studies without any SDoH intervention and studies conducted outside the United States.

### Data extraction

Three authors (S.K., A.K., and S.G.) independently extracted the following data from each article using a standardized form: author, year of publication, target social need, intervention, clinical setting, study design, sample type, and study outcomes. A systematic search of the literature was conducted. Retrieved citations were imported into EndNote X21.2 reference management software for initial title and abstract screening. Studies were evaluated using a hierarchical exclusion process, with titles and abstracts reviewed first to identify studies assessing socioeconomic needs. For records where the title and abstract were insufficient to determine eligibility, full-text articles were retrieved and examined. Two independent reviewers (S.K. and A.K.) evaluated each article to ascertain adherence to all prespecified inclusion criteria. Before initiating data extraction, a standardized protocol was developed by the 3 reviewers (S.K., A.K., and S.G.) to resolve any uncertainties or discrepancies regarding study inclusion or quality assessment. This protocol involved meeting to discuss discrepant texts and achieve consensus or consulting a biostatistician to settle disagreements. Study sponsors were not involved in the development of this protocol.

## Results

A total of 7293 unique records were reviewed (Figure 1). Based on the initial screening using inclusion criteria, full texts were obtained and screened for 2900 records. Of these records, 1522 were excluded as they lacked SDoH interventions or description of SDoH intervention, and 505 were excluded as they were not United States based. Two hundred and ninety-five articles failed to describe any intervention to collect SDoH data (Figure 1). In cases where there was uncertainty about study inclusion or study grade, the paper was discussed until a mutual consensus was reached with the help of a coauthor. Our systematic review identified 78 studies and an additional 20 screening tools and indices screening for adverse social determinants across multiple domains. The most commonly assessed domains included housing instability, food insecurity, transportation barriers, economic stability, and lack of social support. However, studies varied widely in the individual SDoH domains assessed, as well as methods of correlating SDoH data with health outcomes; these studies describe the effectiveness of unique HRSNs, as shown in Figure 1.

### Systematic review findings

The vast majority of tools were missing several key SDoH domains, particularly details regarding race and ethnicity, housing characteristics and type, physical activity, access to healthy food, and environmental exposure. Education was the most common domain across SDoH instruments, followed by healthcare access, transportation, utilities, and housing. Although housing stability was commonly addressed, housing type and characteristics were not widely included. Environmental hazard exposure was relatively poorly covered across the tools, and even when available, its assessment was limited. Many of the tools included in our analysis did not incorporate detailed racial and ethnic data collection. Social connection was included in many tools but was not universal, and the level of stress experienced for any reason was not widely included. Notably, access to healthy food and physical activity was also not covered in most of the tools examined.

While race and ethnicity data may not be routinely captured in all health records, their inclusion in SDoH screening is critical.^13^ Department of Health and Human Services (HHS) policy requires the collection of race/ethnicity data in funded programs to help identify health disparities, ensure nondiscrimination in services, and provide essential information for developing effective health interventions and policies for minority populations.^13,14^ Furthermore, as demonstrated by research on the health consequences of historical redlining, race and ethnicity data are crucial for understanding and addressing the long-term impacts of structural racism on health outcomes.^15^ By consistently collecting this information through SDoH screening, healthcare systems can better identify and address health inequities, tailor interventions to specific communities, and contribute to broader efforts to reverse the effects of historical discrimination in healthcare and social services.^15^

### Development of the proposed SDoH screening tool

The development of our proposed SDoH screening tool was guided by key insights from the systematic review. We made several critical choices in the tool's development:

- Comprehensive domain coverage: Our review revealed that many existing tools lacked coverage in key areas, such as environmental determinants and detailed race/ethnicity data. We ensured our tool included these often-overlooked domains.
- Question selection: We prioritized validated questions from established sources (eg, American Community Survey (ACS), SVI) to ensure reliability and facilitate benchmarking.
- Balance between comprehensiveness and feasibility: While aiming for thorough coverage, we carefully selected questions to provide actionable data without overburdening the screening process.
- EHR integration: Learning from the limitations of paper-based tools, we designed our tool with EHR integration in mind, using standardized response formats and considering workflow implications.
- Alignment with CMS requirements: We ensured our tool meets and exceeds the upcoming CMS mandates for SDoH data collection.
- Actionability: Based on our review findings, we included questions that not only identify social needs but also provide specific information to guide interventions.

The systematic review informed these choices by highlighting gaps in existing tools, identifying best practices in question formulation, and revealing the importance of balancing comprehensiveness with clinical feasibility. For example, our inclusion of environmental determinants was directly informed by the lack of such measures in most existing tools, despite their recognized importance in health outcomes.

### Characteristics of SDoH interventions

The studies included in this systematic review evaluated a diverse range of outcomes related to SDoH interventions. The most commonly assessed outcomes were changes in SDoH status (eg, improvements in housing stability, food security, or social support), health outcomes (eg, changes in physical or mental health status, quality of life, or disease control), healthcare utilization and costs (eg, changes in emergency department visits, hospitalizations, or healthcare expenditures), and process measures (eg, rates of social needs screening, referrals to community resources, or patient satisfaction with SDoH interventions). Some studies also examined provider-level outcomes, such as changes in provider knowledge, attitudes, or practices related to SDoH.

The 20 most commonly used SDoH screening tools and indices (Table 1) identified through additional searches varied widely in their characteristics. Most tools focused on assessing a subset of SDoH domains, such as housing, food, transportation, or interpersonal safety, while some studies employed more comprehensive tools assessing multiple domains (Appendix Table S1). In addition, the tools differed in format, target populations, and integration with clinical workflows, but few studies provided detailed information on their development, validation, or psychometric properties (Appendix Tables S2 and S3).

Education was the most common domain across SDoH instruments, followed by healthcare access, transportation, utilities, and housing (Table 1, Appendix Table S1). Although housing stability was commonly addressed, housing type and characteristics were not widely included. Environmental hazard exposure was relatively poorly covered across the tools, and even when available, its assessment was limited. Many of the tools included in our analysis did not incorporate detailed racial and ethnic data collection. While race and ethnicity are often presumably collected as part of demographic data, even without dedicated SDoH screening, they are rarely captured completely and accurately in real-world scenarios. Social connection was included in many tools but was not universal, and the level of stress experienced for any reason was not widely included. Notably, access to healthy food and physical activity was also not covered in most of the tools examined.

Overall, the heterogeneity in SDoH screening tools and outcome measures underscores the need for greater standardization and validation of SDoH assessment methods to facilitate data harmonization, benchmarking, and intervention evaluation.

Our proposed tool (Table 2) aims to address these gaps by providing a comprehensive, standardized approach to SDoH data collection that covers a wide range of domains and incorporates validated questions from established sources. Table 3 provides a detailed comparison of various aspects of the SDoH domains covered by a set of tools, including our proposed tool. The tools featured in this comparison were chosen based on their validation, standardization, and high potential for clinical integration, as demonstrated by their use in clinical practice settings. These tools were narrowed down from the more extensive list of SDoH screening tools and indices described in Table 1 to facilitate a focused visual comparison of the most relevant and widely used instruments.

**Table 2.** Proposed SDoH screening questionnaire.

Proposed questionnaire
**1. What is your race?** □ White—Print, for example, German, Irish, English, Italian, Lebanese, Egyptian, etc._______ □ Black or African Am.—Print, for example, African American, Jamaican, Haitian, Nigerian, Ethiopian, Somali, etc. ________________________ □ American Indian or Alaska Native—Print name of enrolled or principal tribe(s), for example, Navajo Nation, Blackfeet Tribe, Mayan, Aztec, Native Village of Barrow Inupiat Traditional Government, Nome Eskimo Community, etc. __________________________ □ Chinese □ Vietnamese □ Native Hawaiian □ Filipino □ Korean □ Samoan □ Asian Indian □ Japanese □ Chamorro □ MENA □ Other Asian –Print, for example, Pakistani, Cambodian, Hmong, etc. Some other race—Print race or origin. _______________________
**2. What is your ancestry or ethnic origin?** (For example: Italian, Jamaican, African Am., Cambodian, Cape Verdean, Norwegian, Dominican, French Canadian, Haitian, Korean, Lebanese, Polish, Nigerian, Mexican, Taiwanese, Ukrainian, and so on.) _____________________________
**3. What was your total household income during the PAST 12 MONTHS?** If net income was a loss, enter the amount and mark (X) the “Loss” box next to the dollar amount. □ None or ____dollars □ Loss
**4. What is your highest degree or level of school you have COMPLETED? Mark (X) ONE box. If currently enrolled, mark the previous grade or highest degree received.** NO SCHOOLING COMPLETED □ No schooling completed NURSERY OR PRESCHOOL THROUGH GRADE 12 □ Nursery school □ Kindergarten □ Grade 1 through 11—Specify grade 1-11 __________ □ 12th grade—NO DIPLOMA HIGH SCHOOL GRADUATE □ Regular high school diploma □ GED or alternative credential COLLEGE OR SOME COLLEGE □ Some college credit, but less than 1 year of college credit □ 1 or more years of college credit, no degree □ Associate's degree (for example: AA, AS) □ Bachelor's degree (for example: BA, BS) AFTER BACHELOR'S DEGREE □ Master's degree (for example: MA, MS, MEng, MEd, MSW, MBA) □ Professional degree beyond a bachelor’s degree (for example: MD, DDS, DVM, LLB, JD) □ Doctorate degree (for example: PhD, EdD)
5. **Are you currently employed?** □ YES □ NO **5a) If “yes” to the above, please answer item 5a) below. If “no” to the above, please skip to item** □ Full-time? □ Part-time □ Variable **5b) When did you last work, even for a few days?** □ Within the past 12 months □ 1 to 5 years ago □ Over 5 years ago or never worked
**6. Housing** **6a. What is your housing situation today?** □ I have housing □ I have a place to live today, but I am worried about losing it in the future □ I do not have housing (staying with others, in a hotel, in a shelter, living outside on the street, on a beach, in a car, or in a park) □ I chose not to answer this question **6b. Is this house, apartment, or mobile home—Mark (X) ONE box.** □ Owned by you or someone in this household with a mortgage or loan? Include home equity loans. □ Owned by you or someone in this household without a mortgage or loan? □ Rented? □ Occupied without payment of rent? **6c. What is the monthly mortgage, loan or rent for this house, apartment, or mobile home? ___________** **6d. In the past 12 months, did you find it difficult to pay your monthly rent/mortgage (such as being behind on rent payment or experienced inability to pay rent at all)?** □ YES? □ No **6e. How worried are you right now about not being able to pay your rent, mortgage, or other housing costs?** □ Very worried; □ Moderately worried; □ Not too worried; □ Not worried at all
**7. Which best describes your building? Include all.** □ A building with multiple apartments □ A mobile home □ A one-family house detached from any other house □ A one-family house attached to one or more houses
**8. 8a.How many people are living or staying at your home address? ____________** *INCLUDE everyone who is living or staying here for more than 2 months.* **8b. How many separate bedrooms are in this house, apartment or mobile home?** *If this is an efficiency/studio apartment, print “0”.* Number of bedrooms. ______________
**9. Residence Length** 9a. How long have you been at your current residence? 9b. What is the zip code of your longest-lived residence?
**10. Health Insurance** ***Are you CURRENTLY covered by any of the following types of health insurance or health coverage plans?*** *Mark “Yes” or “No” for EACH type of coverage in items a—h.* **10a. Insurance through a current or former employer or union (of this person or another family member)** □ Yes □ No **10b. Insurance purchased directly from an insurance company (by this person or another family member)** □ Yes □ No **10c. Medicare, for people 65 and older, or people with certain disabilities** □ Yes □ No **10d. Medicaid, Medical Assistance, or any kind of government-assistance plan for those with low incomes or a disability** □ Yes □ No **10e. TRICARE or other military health care**□ Yes □ No **10f. VA (enrolled for VA health care)** □ Yes □ No **10g. Indian Health Service** □ Yes □ No **10h. Any other type of health insurance or health coverage plan**– Specify __________________
**11. At this house, apartment, or mobile home –do you or any member of this household have access to the Internet?** □ Yes □ No
**12. Disability** **12a. Are you deaf or do you have serious difficulty hearing?** □ Yes □ No **12b. Are you blind or do you have serious difficulty seeing, even when wearing glasses?** □ Yes □ No **12c. Because of a physical, mental, or emotional condition, do you have serious difficulty concentrating, remembering, or making decisions? (5 years old or older)** □ Yes □ No **12d. Do you have serious difficulty walking or climbing stairs? (5 years old or older)** □ Yes □ No **12e. Do you have difficulty dressing or bathing? (5 years old or older?** □ Yes □ No **12f. Because of a physical, mental, or emotional condition, do you have difficulty doing errands alone such as visiting a doctor's office or shopping? (15 years old or older)** □ Yes □ No
**13. How well do you speak English?** □ Very well □ Well □ Not well □ Not at all
**14. 14a. What is your marital status?** □ Now married □ Widowed □ Divorced □ Separated □ Never married □ Living with partner **14b. Are you a single parent? (Male/female householder, no spouse or partner present, with own children under 18 years)** □ Yes □ No
**15. Transportation** **15a. How did you usually get to work LAST WEEK? Mark (X) ONE box for the method of transportation used for most of the distance.** □ Car, truck, or van □ Bus □ Subway or elevated rail □ Long-distance train or commuter rail □ Light rail, streetcar, or trolley □ Ferryboat □ Taxicab □ Motorcycle □ Bicycle □ Walked □ Worked from home □ Other method **15b. In the past 12 months, has lack of reliable transportation kept you from medical appointments, meetings, work, or from getting things needed for daily living?** □ Yes □ No
**16**. **In the past year, have you or any family members you live with been unable to get any of the following when it was really needed? Check all that apply.** Food □ Yes □ No Healthy food (fresh fruits and vegetables) □ Yes □ No Medicines or Healthcare (Medical, Dental, Mental Health, Vision) □ Yes □ No Phone □ Yes □ No I choose not to answer this question □ If you responded “yes” to any of the above, please answer the following questions: DURING THE PAST 12 MONTHS, please indicate if you or anyone in the family had problems paying or were unable to pay any medical bills?^16^ □ YES? □ NO? DURING THE PAST 12 MONTHS, has medical care been delayed because of worry about the cost?^16^ □ YES? □ NO? How worried are you right now about not being able to pay your rent, mortgage, or other housing costs?^16^ □ Very worried; □ Moderately worried; □ Not too worried; □ Not worried at all How worried are you right now about not being able to pay your normal monthly bills?^16^ □ Very worried; □ Moderately worried; □ Not too worried; □ Not worried at all
17. **How often do you see or talk to people that you care about and feel close to? (For example: talking to friends on the phone, visiting friends or family, going to church or club meetings)** □ Less than once a week □ 1 0r 2 times a week □ 3 to 5 times a week □ 6 or more times a week □ I choose not to answer this question
**18. How much do you agree or disagree with the following statements about your neighborhood? Would you say… There are people I can count on in this neighborhood:** □ Definitely agree; □ Somewhat agree; □ Somewhat disagree; □ Definitely disagree
**19. 19a.Over the last 2 weeks, how often have you been bothered by the following problems?** a. Little interest or pleasure in doing things. b. Feeling down, depressed or hopeless □ Not at all (0) □ Several days (1) □ More than half the days(2) □ Nearly every day(3) Total score for 19a + 19b If the score is 3 or greater, major depressive disorder is likely. **19b. How often does your partner?** A. Physically hurt you? B. Insult you or talk down to you? C. Threaten you with harm? D. Scream or curse at you? 5-point Likert scale: never (1 point)→rarely (2); sometimes (3); fairly often (4); frequently (5); Scores ≥10.5 are positive For Spanish version, cutoff score = 5.5
**20. Physical activity** **20a.In the last 30 days, other than the activities you did for work, on average, how many days per week did you engage in moderate exercise (like walking fast, running, jogging, dancing, swimming, biking, or other similar activities)?** □ 0 □ 1 □ 2 □ 3 □ 4 □ 5 □ 6 □ 7 **20b. On average, how many minutes did you usually spend exercising at this level on one of those days?** □ 0 □ 10 □ 20 □ 30 □ 40 □ 50 □ 60 □ 90 □ 120 □ 150 or greater Follow these 2 steps to decide if the person has a physical activity need: Calculate [“number of days” selected] × [“number of minutes” selected] = [number of minutes of exercise per week]. Apply the right age threshold: Under 6 years old: there are no physical activity standards for children under 6 years of age. Age 6 to 17: Less than an average of 60 minutes a day shows an HRSN. Age 18 or older: Less than 150 minutes a week shows an HRSN.

**Table 3.** Comparison of various aspects of social determinants of health among the tools.

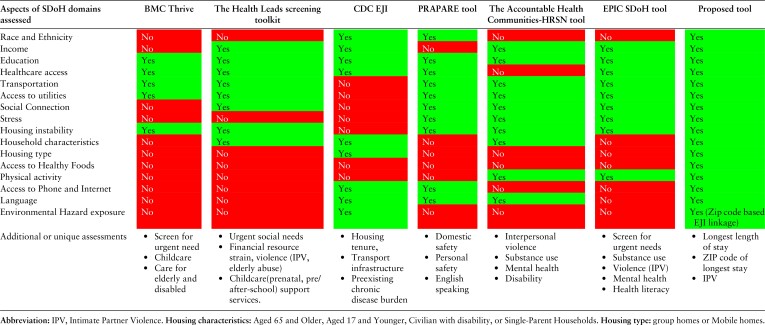

## Discussion

### Main findings

Our systematic review found consistent evidence linking adverse social determinants with worse health outcomes across a range of domains. Housing instability, food insecurity, transportation barriers, financial hardship, and lack of social support were among the most frequently identified SDoH factors associated with negative health impacts. Studies measuring multiple or >4 SDoH factors were 32% (25/78 studies); stress was measured in 30% of the studies (24/78); socioeconomic factors including employment, education, and income were measured in 20% of the studies (16/78); access to food, utilities, and healthcare were measured in 12.8% of the studies (10/78); household characteristics were measured in 3.8% of the studies (3/78); transport in 2.5% of the studies (2/78); minority and social connection were measured in 1.2% of the studies (1/78). These findings underscore the importance of assessing and addressing SDoH in clinical settings to improve patient outcomes and reduce health disparities.

Our study revealed significant heterogeneity in both the content and implementation of these instruments. This variability manifests in several ways. First, many popular tools are missing critical SDoH domains, such as detailed race/ethnicity data and access to healthy food, and environmental determinants are often overlooked.^16^ Second, even when tools assess the same domains, they often do so in different, noncomparable ways.^16^ This inconsistency makes it challenging to aggregate data across healthcare systems, conduct meaningful population-level analyses, or benchmark performance in addressing social needs.^16^

### Why screen?

SDoH significantly shape individuals’ understanding, beliefs, skills, and health-related actions.^17^ HRSN refer to individual barriers shaped by social determinants that impact health and well-being, such as food insecurity, inadequate housing, and lack of transportation.^18^ SDoH represent upstream socioeconomic conditions that shape HRSN, such as poverty, racism, and inequitable access to resources. SDoH tools also include access to monetary and social support resources, as well as critical healthcare services.^17^ SDoH tools are broad environmental conditions affecting health outcomes across 5 domains: social context, economic stability, education, neighborhood environment, and healthcare access. SDoH tools assess these population-level factors.^18^ While there is significant overlap between SDoH and HRSN, particularly in domains like housing, food security, and transportation, SDoH tools often include broader measures of community-level factors that may not be immediately actionable at the individual level.^18^ For instance, a SDoH tool might assess neighborhood safety or air quality, while an HRSN tool would focus on whether an individual has immediate safety concerns in their home or lacks transportation to medical appointments.^18^ SDoH can impact everyone and can be positive or negative, while HRSN typically reflect specific challenges experienced by individuals due to their circumstances. In addition, economic instability can result in patients postponing or neglecting medical appointments and medication.^17^ Moreover, insufficient access to nutritious food or safe, healthy environments can impede adherence to treatments that necessitate lifestyle and behavioral modifications.^17^ SDoH are increasingly recognized as upstream drivers of recurring hospitalizations, diminished quality of life, and persistent sociodemographic disparities in mortality rates.^17^ Additionally, shared decision-making, which integrates patient-centered care principles and inherently prioritizes each patient's preferences, values, and needs in all medical choices, can be heavily influenced by SDoH (Appendix Table S2).^17^ Our proposed tool aims to bridge these approaches by incorporating both SDoH and HRSN elements. This comprehensive approach allows for a more nuanced understanding of both individual needs and the broader social context influencing health outcomes. By doing so, our tool provides actionable data for immediate interventions while also informing broader population health strategies and policy initiatives.

SDoH data collection at the point of care can help improve health systems’ understanding of patients’ outstanding social needs and identify inequities that influence the length of hospitalization, healthcare utilization, hospital readmission, and adverse outcomes.^19^ The resulting knowledge may inform much-needed efforts to deliver patient-centered care and improve patient and population outcomes. Social needs phenotyping allows for understanding the specific unfavorable social determinants experienced by the patient and helps identify resources to address them.^20^ It is crucial to have tools that can assess and identify social risk to help identify socially vulnerable patient populations, design collaborative interventions to connect them with available resources, and guide primary and secondary prevention efforts to reduce glaring disparities in morbidity and mortality.^20^

### Who to screen?

Based on the reviewed literature, there is growing evidence supporting universal screening of individual-level SDoH across all populations, rather than targeting specific subgroups.^21^ Multiple studies have demonstrated that SDoH impact health outcomes across diverse demographics, suggesting that universal screening may be more effective in revealing important SDoH needs and reducing potential stigma associated with targeted approaches.^21^ Furthermore, research indicates that complementing individual-level SDoH screening with area-based metrics can be valuable in identifying communities experiencing high social deprivation, food insecurity, or limited healthcare access, which may benefit from more intensive or specialized screening efforts.^22,23^ For example, neighborhoods with high poverty rates may warrant enhanced efforts to assess food and housing insecurity among residents, and areas with low physician density may benefit from telemedicine approaches.^24,25^ It is known that marginalized racial/ethnic subgroups experience a high risk of structural racism and discrimination, which directly and indirectly influences access to critical resources and affects SDoH across domains, with resulting health effects.^26^ Variation in experienced social disadvantage across racial and ethnic subgroups and downstream effects on cardiovascular disease—the #1 cause of mortality in the United States—has been reported previously.^26,27^ Efforts to screen for SDoH should be guided by cultural humility and pay close attention to the unique social risks experienced by historically underserved racial and ethnic populations.^28^

### When to screen?

Screening should occur at periodic visits like annual wellness examinations and new patient intakes, facilitating screening integration into routine workflows and clinical decision support systems.^29^ Hospitalizations, emergency department visits, and urgent care visits represent opportune times when someone interacting with the health system for an acute issue can have any recently emerged SDoH needs identified.^30^ In particular, in-patient stays allow sufficient time for detailed SDoH screening.^30^ This can help identify SDoH needs among patients at the highest risk for adverse outcomes, such as readmissions, for whom SDoH interventions may significantly reduce the disease burden.^30^ More frequent in-person or virtual screening may be warranted for high-risk patients.

### What to screen for?

The CMS letter to state officials discusses several SDoH domains that can be addressed through Medicaid and CHIP programs.^31,32^ The agency also developed an HRSN screening checklist that can guide the development and validation of similar screening tools in diverse patient populations.^32^ Some of the key SDoH domains included in the documents are shown in Figure 1.

The CMS document also provides examples of how services and support in these areas can be covered through Medicaid and CHIP to address social needs and improve health.^32^ Our tool contains a mix of HRSN screening questions to identify individual-level social needs as well as measures of SDoH factors, including socioeconomic status, environmental risks, and healthcare access.

### What to do with the information?

Studies suggest that the Accountable Health Community (AHC) model, supported by CMS, has effectively assessed the HRSNs of eligible beneficiaries and facilitated access to community services, leading to a significant decrease in emergency department visits.^33^ However, interviews with AHC model staff, community service providers, and beneficiaries have revealed significant obstacles in connecting beneficiaries with the necessary services to address the identified social risk factors.^22^ Furthermore, when connections were established, it became apparent that the available resources were often inadequate to address the diverse social needs of the beneficiaries.^22^ To ensure the successful linkage of individual patients with community services, it is essential to identify “community champions” who may act as a bridge between the health system and the patients. Health systems should pay close attention to *voices from the community* and plan for additional resource investments to guarantee an adequate supply of services, which may be crucial for effectively assisting beneficiaries in their communities.^22,33^ With limited resources, accurate identification of actionable issues by the screening tool becomes crucial to maximizing the utilization of community resources. Without this, there may be further harm in screening and highlighting adverse SDoH without clear ways to address the identified social factor. There is also the potential to overwhelm already strained health and social service systems if identified needs cannot be adequately addressed.

In this article, we compare various SDoH tools and propose a harmonized tool that we believe would help optimize the collection of the required information in a comprehensive, culturally sensitive fashion and provide actionable information that may help improve health outcomes and mitigate health inequities. In opting for a more comprehensive tool, we aimed to balance the depth and actionability of screening on priority domains with the need for broad surveillance and standardized data collection. Though a targeted approach may be better suited to certain contexts, we prioritized inclusiveness across major social determinants. The rationale for comprehensive SDoH screening and the considerations for who to screen, when to screen, what to screen for, and what to do with the information are discussed in the Supplementary File.

### Environmental determinants of health

Environmental factors like pollution, hazardous waste, heat, and access to green space interact with SDoH like housing and neighborhood safety disproportionately affect marginalized groups. Incorporating questions about environmental exposures into patient screening can help clinicians understand patients’ overall disease risk.^34,35^ This allows for treatment plans that mitigate climate health risks and address health disparities stemming from environmental injustice. Environmental Justice Index (EJI) (an aggregate of census tract level environmental exposures and social vulnerability) has been associated with increasing prevalence of adverse health-related outcomes, in particular, cardiometabolic outcomes.^36^ However, ironically, despite this recognition of the crucial role of the environment, most individual patient-level SDoH tools do not include information regarding environmental exposure.

We have given particular attention to environmental determinants of health in our proposed tool, dedicating a full sub-category to this domain. This emphasis is justified by the growing body of evidence linking environmental factors to health outcomes, as well as the consistent neglect of this domain in existing tools.^36^ environmental determinants, including air and water quality, exposure to toxins, and climate-related health risks, have profound impacts on health, often disproportionately affecting vulnerable populations.^36^ By incorporating this often-overlooked domain, our tool provides a more comprehensive assessment of the factors influencing patient health.^36^ A post hoc analysis of SPRINT trial has shown that the patients exposed to high levels of air pollution had higher reduction in cardiovascular outcomes when placed on intense vs standard antihypertensive therapy.^37^

### Proposed ToolArt of framing questions and characteristics of an optimal SDoH screening tool

When formulating SDoH screening questions, clinicians must consider patients’ perspectives on sensitive information collection using unbiased language.^10,11^ Studies show answering such questions involves multiple steps like interpretation and judgment formation, with variation across groups often stemming from response editing tendencies and question interpretation leading to social desirability bias.^10,11^ Resulting data inconsistencies underscore the need for neutral phrasing.^12^ Clinicians should also receive training on historical marginalization and sensitive communication to mitigate biases. SDoH data must also be collected longitudinally to assess temporal variations in risk and intervention effects.^10^ Concise, validated surveys are preferred, though they face a tradeoff between brevity and nuance.^10^ Our proposed screening tool comprises questions from previously validated instruments to enable reliable, reproducible social determinants measurement, as shown in Table 2.^13^ Further details on the art of framing questions and characteristics of an optimal SDoH screening tool are provided in the Supplementary File.

**Figure 2.**
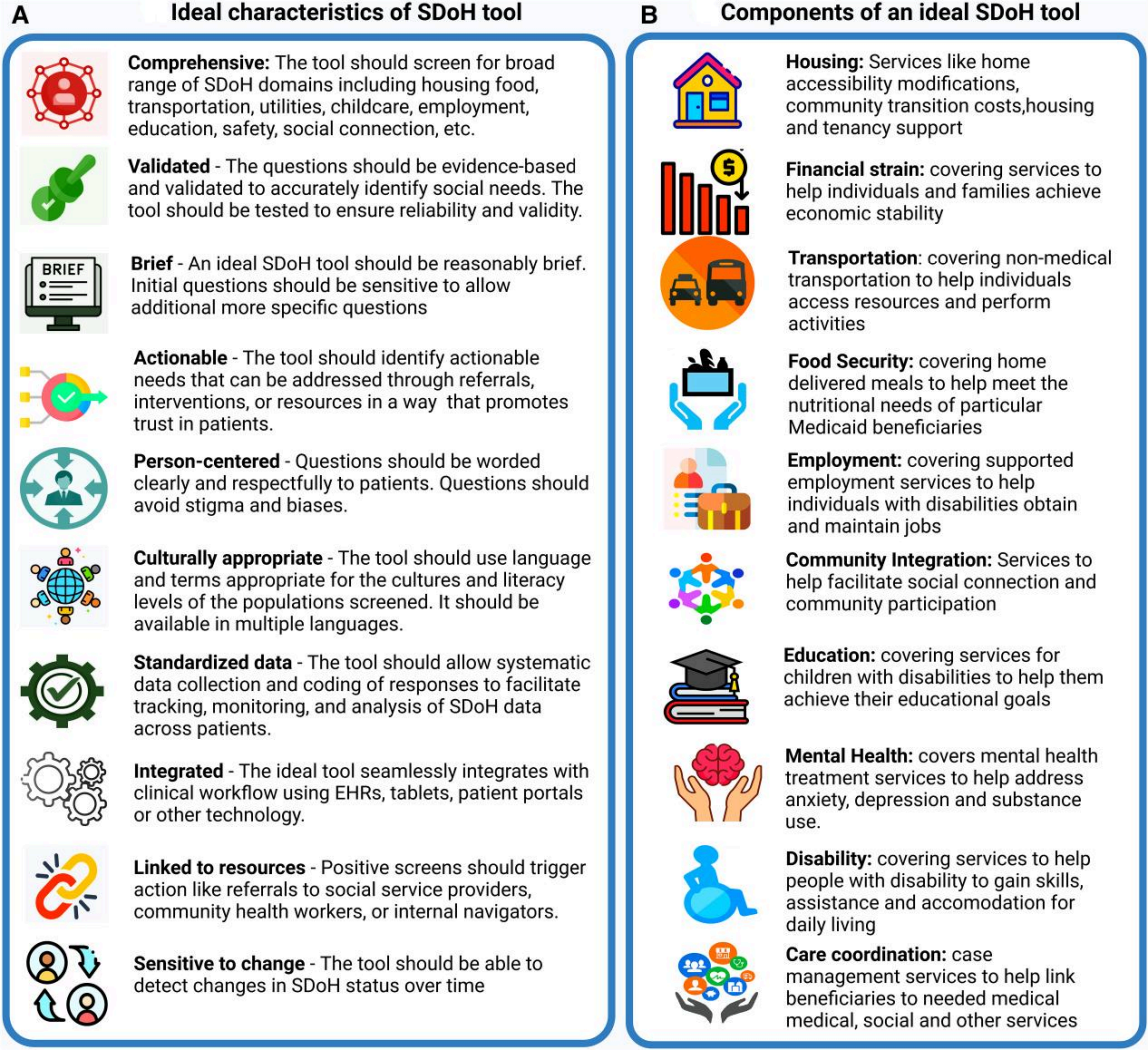
Characteristics and components of ideal SDoH screening tool.

Standardization of SDoH screening tools is critical so that populations and intervention programs can be used as benchmarks for implementation. Standardization also allows for comparing populations and helps allocate resources. The ideal SDoH screening tool is brief yet comprehensive, validated, actionable, person-centered, culturally appropriate, integrated into the workflow, and linked to intervention resources, as shown in Figure 2.

Our proposed comprehensive SDoH screening tool (Table 2) incorporates questions from validated sources to enable reliable, reproducible social determinants measurement across a wide range of domains. The choice of specific domains and measures aimed to optimize validity, feasibility, and linkage to health impacts based on prior evidence (Appendix Table S4). Incorporating questions from the ACS and SVI and EJI allows for building individual-level social vulnerability and environmental justice metrics, metrics allow for benchmarking and policy integration (Table 3).

Comprehensive and inclusive race and ethnicity data collection is crucial in SDoH screening tools. Many tools assume this information is already captured in EHRs, but it is often incomplete or missing options for several minority groups. Aggregating data into broad categories can obscure important disparities, such as those within Asian American subgroups. Our proposed SDoH screening tool aims to capture detailed race and ethnicity information, including major Asian American, Native Hawaiian, Pacific Islander, and Middle Eastern/North African (MENA) subgroups, to enable granular analyses and targeted interventions addressing health disparities. The lack of disaggregated data during the COVID-19 pandemic masked the disproportionate impact on Asian American communities and led to their omission from priority groups in vaccine allocation reports, potentially affecting resource access.

Notably, our tool offers unique features compared with other widely used SDoH screening tools, including an assessment of physical activity levels, a question regarding lifetime environmental exposure for extrapolating an individual-level EJI, and a comprehensive evaluation of housing characteristics, housing type, and access to healthy foods (Table 3). The tool also intentionally collects detailed race and ethnicity information, including options for major subgroups, to facilitate granular analyses and targeted interventions that avoid masking disparities. By providing a holistic assessment of SDoH across multiple domains, our tool aims to enable standardized data collection, risk stratification, and focused initiatives to address health inequities at both individual and population levels.

## Future directions

Future research should focus on developing, validating, and implementing optimal universal SDoH screening tools integrated into clinical workflows, treating social and clinical risks equally. Enriching individual SDoH data with administrative codes and residential addresses can identify individuals needing detailed SDoH phenotyping. Capturing SDoH data as retrievable EHR variables using LOINC codes, SNOMED codes, and HL7 FHIR standards is crucial, along with incentivizing SDoH Z codes utilization. Machine learning and artificial intelligence tools, particularly natural language processing, can capture and structure SDoH data from unstructured EHR notes for risk stratification, population health management, and clinical decision support. Investigating the impact of SDoH interventions on clinical outcomes through novel polysocial indices and risk scores is essential. Aggregated, de-identified SDoH data at the population level could guide public health initiatives addressing social and environmental health disparities. Logistical and technical challenges limiting such integration should be addressed.

In addition, the concept of a “polysocial risk score” (pSRS) represents an innovative approach to quantifying the cumulative impact of multiple social determinants on cardiovascular health risks.^38^ Similar to polygenic risk scores in genetics, a pSRS aggregates data from various SDoH domains to create a comprehensive measure of social risk.^38^ Recent evidence suggests pSRS can improve CVD risk prediction beyond traditional clinical factors.^38^ For example, Javed et al. developed a pSRS for atherosclerotic cardiovascular disease using national survey data that included 7 key SDoH factors and demonstrated improved model discrimination when added to traditional risk factors.^38^ Hong and Mainous created a county-level pSRS that outperformed existing indices in predicting CVD prevalence.^39^ In the clinical setting, Huang et al. used electronic health record data to develop a pSRS for hospitalization risk in patients with type 2 diabetes, explaining over 33% of the variation in risk after adjusting for clinical factors.^40^ Implementation of pSRS in practice would require robust validation across diverse populations, standardization across healthcare systems, and careful integration with existing clinical workflows.^40^ However, the potential for more precise, personalized, and proactive cardiovascular care based on comprehensive social risk assessment is significant.^38^ Future research should focus on developing pSRS using longitudinal cohorts, testing performance in diverse subgroups, and evaluating real-world clinical impact when integrated into decision support systems.

Standardizing SDoH-related risk across diverse communities and varying levels of deprivation is critical for the widespread application of pSRS. Several approaches show promise in this regard. Relative risk scaling, similar to techniques used in polygenic risk scores, could adjust SDoH factors based on their distribution within specific populations.^38,41^ Multi-level modeling can account for both individual and community-level SDoH data, reflecting the nested nature of social risks.^42^ Bayesian methods offer the potential to integrate prior knowledge about SDoH–health relationships, while machine learning algorithms can capture complex, nonlinear associations between multiple SDoH factors and health outcomes.^43^ Establishing standardized reference populations for different community types (eg, urban, rural, and suburban) could further enhance comparability across diverse settings.^44^ While implementing these techniques would require large-scale, diverse datasets and cross-institutional collaboration, such standardization efforts are crucial for enhancing the comparability and actionability of SDoH data in cardiovascular risk prediction and management across varied populations and geographic areas.

## Limitations

This study has several limitations. First, the proposed screening tool has not yet been validated or tested for feasibility and acceptability. However, individual questions have been derived from validated tools without any adaptations. Second, the tool contains a mix of HRSNs and SDoH measures, which may make comparisons to more targeted HRSN instruments more challenging Finally, our analysis relied on the content domains covered by each tool rather than comprehensive comparative data on validity, implementation outcomes, or impacts on disparities. More research is required to determine if this tool has greater sensitivity, specificity and psychometrics in capturing actionable data on social risks vs other available instruments. Despite these limitations, consolidation of key domains from validated HRSN and SDoH tools may provide an efficient approach to initial screening. However, real-world evidence is still needed to substantiate the hypothesized benefits of this tool over existing instruments.

The case for care delivery organizations to adopt our proposed tool is compelling, particularly given the current healthcare landscape. First, our tool addresses the gaps in domain coverage identified in our review, providing a more comprehensive assessment of patients’ social needs. Second, the standardization offered by our tool facilitates data aggregation and comparison across healthcare systems. Third, our tool is designed for seamless integration into electronic health records, addressing a key barrier to widespread adoption of SDoH screening.

The timing for adopting a new, standardized SDoH screening tool is particularly opportune. With the new CMS mandates for SDoH data collection, healthcare organizations are actively seeking solutions that not only meet regulatory requirements but also provide actionable insights for improving patient care. Our tool is positioned to meet both these needs.

Finally, the importance of standardized data infrastructure in healthcare cannot be overstated. As we move toward more integrated, data-driven healthcare systems, having a consistent approach to collecting and analyzing SDoH data becomes crucial. Our tool contributes to building this standardized infrastructure, enabling more effective collaboration between healthcare providers, community organizations, and policymakers in addressing SDoH.

## Conclusion

Standardized SDoH screening tools are needed to identify at-risk patients, guide interventions, and track progress toward health equity. Our proposed tool offers a comprehensive approach to SDoH assessment, but further validation and comparative effectiveness research is needed to optimize its implementation and impact. Integrating SDoH data into clinical workflows and research databases remains a critical challenge and opportunity for improving population health and reducing disparities.

## Contribution statement

All authors participated in the research and preparation of the manuscript as per the International Committee of Medical Journal Editors (ICMJE).

## Supplementary Material

qxae151_Supplementary_Data
