# Supplementary material for: Standardizing social determinants of health data: a proposal for a comprehensive screening tool to address health equity a systematic review
Source: Health Aff Sch. 2024 Nov 14;2(12):qxae151. doi: 10.1093/haschl/qxae151 (PMC11642620; doi:10.1093/haschl/qxae151)
Supplement: qxae151_Supplementary_Data [file qxae151_supplementary_data.zip › References.docx]

**References**

45. Tomita A, Herman DB. The role of a critical time intervention on the experience of continuity of care among persons with severe mental illness after hospital discharge. *J Nerv Ment Dis*. Jan 2015;203(1):65-70. doi:10.1097/nmd.0000000000000224

46. Garg A, Toy S, Tripodis Y, Silverstein M, Freeman E. Addressing social determinants of health at well child care visits: a cluster RCT. *Pediatrics*. Feb 2015;135(2):e296-304. doi:10.1542/peds.2014-2888

47. Kangovi S, Mitra N, Norton L, et al. Effect of Community Health Worker Support on Clinical Outcomes of Low-Income Patients Across Primary Care Facilities: A Randomized Clinical Trial. *JAMA Intern Med*. Dec 1 2018;178(12):1635-1643. doi:10.1001/jamainternmed.2018.4630

48. Mendelsohn AL, Valdez PT, Flynn V, et al. Use of videotaped interactions during pediatric well-child care: impact at 33 months on parenting and on child development. *J Dev Behav Pediatr*. Jun 2007;28(3):206-12. doi:10.1097/DBP.0b013e3180324d87

49. Klevens J, Kee R, Trick W, et al. Effect of screening for partner violence on women's quality of life: a randomized controlled trial. *Jama*. Aug 15 2012;308(7):681-9. doi:10.1001/jama.2012.6434

50. Silverstein M, Mack C, Reavis N, Koepsell TD, Gross GS, Grossman DC. Effect of a Clinic-Based Referral System to Head StartA Randomized Controlled Trial. *JAMA*. 2004;292(8):968-971. doi:10.1001/jama.292.8.968

51. Herman DB, Conover S, Gorroochurn P, Hinterland K, Hoepner L, Susser ES. Randomized trial of critical time intervention to prevent homelessness after hospital discharge. *Psychiatr Serv*. Jul 2011;62(7):713-9. doi:10.1176/ps.62.7.pss6207_0713

52. Tomita A, Herman DB. The impact of critical time intervention in reducing psychiatric rehospitalization after hospital discharge. *Psychiatr Serv*. Sep 1 2012;63(9):935-7. doi:10.1176/appi.ps.201100468

53. Garg A, Butz AM, Dworkin PH, Lewis RA, Thompson RE, Serwint JR. Improving the management of family psychosocial problems at low-income children's well-child care visits: the WE CARE Project. *Pediatrics*. Sep 2007;120(3):547-58. doi:10.1542/peds.2007-0398

54. Gottlieb LM, Hessler D, Long D, et al. Effects of Social Needs Screening and In-Person Service Navigation on Child Health: A Randomized Clinical Trial. *JAMA Pediatr*. Nov 7 2016;170(11):e162521. doi:10.1001/jamapediatrics.2016.2521

55. Waitzkin H, Getrich C, Heying S, et al. Promotoras as mental health practitioners in primary care: a multi-method study of an intervention to address contextual sources of depression. *J Community Health*. Apr 2011;36(2):316-31. doi:10.1007/s10900-010-9313-y

56. Bronstein LR, Gould P, Berkowitz SA, James GD, Marks K. Impact of a Social Work Care Coordination Intervention on Hospital Readmission: A Randomized Controlled Trial. *Soc Work*. Jul 2015;60(3):248-55. doi:10.1093/sw/swv016

57. Berkowitz SA, Hulberg AC, Hong C, et al. Addressing basic resource needs to improve primary care quality: a community collaboration programme. *BMJ Qual Saf*. Mar 2016;25(3):164-72. doi:10.1136/bmjqs-2015-004521

58. Sege R, Preer G, Morton SJ, et al. Medical-Legal Strategies to Improve Infant Health Care: A Randomized Trial. *Pediatrics*. Jul 2015;136(1):97-106. doi:10.1542/peds.2014-2955

59. Feigelman S, Dubowitz H, Lane W, Grube L, Kim J. Training pediatric residents in a primary care clinic to help address psychosocial problems and prevent child maltreatment. *Acad Pediatr*. Nov-Dec 2011;11(6):474-80. doi:10.1016/j.acap.2011.07.005

60. Morales ME, Berkowitz SA. The Relationship between Food Insecurity, Dietary Patterns, and Obesity. *Curr Nutr Rep*. Mar 2016;5(1):54-60. doi:10.1007/s13668-016-0153-y

61. Dubowitz H, Lane WG, Semiatin JN, Magder LS, Venepally M, Jans M. The safe environment for every kid model: impact on pediatric primary care professionals. *Pediatrics*. Apr 2011;127(4):e962-70. doi:10.1542/peds.2010-1845

62. Dubowitz H, Lane WG, Semiatin JN, Magder LS. The SEEK model of pediatric primary care: can child maltreatment be prevented in a low-risk population? *Acad Pediatr*. Jul-Aug 2012;12(4):259-68. doi:10.1016/j.acap.2012.03.005

63. Krieger JW, Takaro TK, Song L, Weaver M. The Seattle-King County Healthy Homes Project: a randomized, controlled trial of a community health worker intervention to decrease exposure to indoor asthma triggers. *Am J Public Health*. Apr 2005;95(4):652-9. doi:10.2105/ajph.2004.042994

64. Hassan A, Scherer EA, Pikcilingis A, et al. Improving Social Determinants of Health: Effectiveness of a Web-Based Intervention. *American Journal of Preventive Medicine*. 2015/12/01/ 2015;49(6):822-831. doi:https://doi.org/10.1016/j.amepre.2015.04.023

65. Haas JS, Linder JA, Park ER, et al. Proactive tobacco cessation outreach to smokers of low socioeconomic status: a randomized clinical trial. *JAMA Intern Med*. Feb 2015;175(2):218-26. doi:10.1001/jamainternmed.2014.6674

66. Becker MG, Hall JS, Ursic CM, Jain S, Calhoun D. Caught in the Crossfire: the effects of a peer-based intervention program for violently injured youth. *J Adolesc Health*. Mar 2004;34(3):177-83. doi:10.1016/j.jadohealth.2003.04.001

67. Dicker RA, Jaeger S, Knudson MM, et al. Where Do We Go From Here? Interim Analysis to Forge Ahead in Violence Prevention. *Journal of Trauma and Acute Care Surgery*. 2009;67(6)

68. Juillard C, Cooperman L, Allen I, et al. A decade of hospital-based violence intervention: Benefits and shortcomings. *Journal of Trauma and Acute Care Surgery*. 2016;81(6)

69. Garg A, Marino M, Vikani AR, Solomon BS. Addressing families' unmet social needs within pediatric primary care: the health leads model. *Clin Pediatr (Phila)*. Dec 2012;51(12):1191-3. doi:10.1177/0009922812437930

70. Paris R. "For the dream of being here, one sacrifices...": voices of immigrant mothers in a home visiting program. *Am J Orthopsychiatry*. Apr 2008;78(2):141-51. doi:10.1037/0002-9432.78.2.141

71. Garg A, Sarkar S, Marino M, Onie R, Solomon BS. Linking urban families to community resources in the context of pediatric primary care. *Patient Educ Couns*. May 2010;79(2):251-4. doi:10.1016/j.pec.2009.10.011

72. Rodabaugh KJ, Hammond M, Myszka D, Sandel M. A medical-legal partnership as a component of a palliative care model. *J Palliat Med*. Jan 2010;13(1):15-8. doi:10.1089/jpm.2009.0203

73. Ryan AM, Kutob RM, Suther E, Hansen M, Sandel M. Pilot study of impact of medical-legal partnership services on patients' perceived stress and wellbeing. *J Health Care Poor Underserved*. Nov 2012;23(4):1536-46. doi:10.1353/hpu.2012.0179

74. Weintraub D, Rodgers MA, Botcheva L, et al. Pilot study of medical-legal partnership to address social and legal needs of patients. *J Health Care Poor Underserved*. May 2010;21(2 Suppl):157-68. doi:10.1353/hpu.0.0311

75. O'Sullivan MM, Brandfield J, Hoskote SS, et al. Environmental improvements brought by the legal interventions in the homes of poorly controlled inner-city adult asthmatic patients: a proof-of-concept study. *J Asthma*. Nov 2012;49(9):911-7. doi:10.3109/02770903.2012.724131

76. Cohen AJ, Richardson CR, Heisler M, et al. Increasing Use of a Healthy Food Incentive: A Waiting Room Intervention Among Low-Income Patients. *Am J Prev Med*. Feb 2017;52(2):154-162. doi:10.1016/j.amepre.2016.11.008

77. Beck AF, Henize AW, Kahn RS, Reiber KL, Young JJ, Klein MD. Forging a pediatric primary care-community partnership to support food-insecure families. *Pediatrics*. Aug 2014;134(2):e564-71. doi:10.1542/peds.2013-3845

78. Needlman R, Toker KH, Dreyer BP, Klass P, Mendelsohn AL. Effectiveness of a primary care intervention to support reading aloud: a multicenter evaluation. *Ambul Pediatr*. Jul-Aug 2005;5(4):209-15. doi:10.1367/a04-110r.1

79. Mendelsohn AL, Mogilner LN, Dreyer BP, et al. The impact of a clinic-based literacy intervention on language development in inner-city preschool children. *Pediatrics*. Jan 2001;107(1):130-4. doi:10.1542/peds.107.1.130

80. Seligman HK, Lyles C, Marshall MB, et al. A Pilot Food Bank Intervention Featuring Diabetes-Appropriate Food Improved Glycemic Control Among Clients In Three States. *Health Aff (Millwood)*. Nov 2015;34(11):1956-63. doi:10.1377/hlthaff.2015.0641

81. Coker AL, Follingstad D, Garcia LS, Williams CM, Crawford TN, Bush HM. Association of intimate partner violence and childhood sexual abuse with cancer-related well-being in women. *J Womens Health (Larchmt)*. Nov 2012;21(11):1180-8. doi:10.1089/jwh.2012.3708

82. Sanders LM, Gershon TD, Huffman LC, Mendoza FS. Prescribing Books for Immigrant Children: A Pilot Study to Promote Emergent Literacy Among the Children of Hispanic Immigrants. *Archives of Pediatrics & Adolescent Medicine*. 2000;154(8):771-777. doi:10.1001/archpedi.154.8.771

83. Silverstein M, Iverson L, Lozano P. An English-language clinic-based literacy program is effective for a multilingual population. *Pediatrics*. May 2002;109(5):E76-6. doi:10.1542/peds.109.5.e76

84. Krasnoff M, Moscati R. Domestic violence screening and referral can be effective. *Ann Emerg Med*. Nov 2002;40(5):485-92. doi:10.1067/mem.2002.128872

85. Short LM, Hadley SM, Bates B. Assessing the success of the WomanKind program: an integrated model of 24-hour health care response to domestic violence. *Women Health*. 2002;35(2-3):101-19. doi:10.1300/J013v35n02_07

86. McCaw B, Berman WH, Syme SL, Hunkeler EF. Beyond screening for domestic violence: a systems model approach in a managed care setting. *Am J Prev Med*. Oct 2001;21(3):170-6. doi:10.1016/s0749-3797(01)00347-6

87. Gillum TL, Sun CJ, Woods AB. Can a health clinic-based intervention increase safety in abused women? Results from a pilot study. *J Womens Health (Larchmt)*. Aug 2009;18(8):1259-64. doi:10.1089/jwh.2008.1099

88. Teufel JA, Brown SL, Thorne W, Goffinet DM, Clemons L. Process and Impact Evaluation of a Legal Assistance and Health Care Community Partnership. *Health Promotion Practice*. 2009;10(3):378-385.

89. Ulbrich PM, Stockdale J. Making family planning clinics an empowerment zone for rural battered women. *Women Health*. 2002;35(2-3):83-100. doi:10.1300/J013v35n02_06

90. Zachary MJ, Schechter CB, Kaplan ML, Mulvihill MN. Provider evaluation of a multifaceted system of care to improve recognition and management of pregnant women experiencing domestic violence. *Women's Health Issues*. 2002/01/01/ 2002;12(1):5-15. doi:https://doi.org/10.1016/S1049-3867(01)00142-6

91. Smith S, Malinak D, Chang J, et al. Implementation of a food insecurity screening and referral program in student-run free clinics in San Diego, California. *Prev Med Rep*. Mar 2017;5:134-139. doi:10.1016/j.pmedr.2016.12.007

92. Pettignano R, Caley SB, Bliss LR. Medical-legal partnership: impact on patients with sickle cell disease. *Pediatrics*. Dec 2011;128(6):e1482-8. doi:10.1542/peds.2011-0082

93. Nguyen AL, Angulo M, Haghi LL, et al. A clinic-based pilot intervention to enhance diabetes management for elderly Hispanic patients. *J Health Environ Educ*. 2016;8:1-6. doi:10.18455/08001

94. Onyekere C, Ross S, Namba A, Ross JC, Mann BD. Medical Student Volunteerism Addresses Patients' Social Needs: A Novel Approach to Patient-Centered Care. *Ochsner J*. Spring 2016;16(1):45-9.

95. Pettignano R, Caley SB, McLaren S. The health law partnership: adding a lawyer to the health care team reduces system costs and improves provider satisfaction. *J Public Health Manag Pract*. Jul-Aug 2012;18(4):E1-3. doi:10.1097/PHH.0b013e31823991a9

96. Beck AF, Klein MD, Schaffzin JK, Tallent V, Gillam M, Kahn RS. Identifying and treating a substandard housing cluster using a medical-legal partnership. *Pediatrics*. Nov 2012;130(5):831-8. doi:10.1542/peds.2012-0769

97. Klein MD, Beck AF, Henize AW, Parrish DS, Fink EE, Kahn RS. Doctors and lawyers collaborating to HeLP children--outcomes from a successful partnership between professions. *J Health Care Poor Underserved*. Aug 2013;24(3):1063-73. doi:10.1353/hpu.2013.0147

98. Zheng DJ, Shyr D, Ma C, Muriel AC, Wolfe J, Bona K. Feasibility of systematic poverty screening in a pediatric oncology referral center. *Pediatr Blood Cancer*. Dec 2018;65(12):e27380. doi:10.1002/pbc.27380

99. Kurani SS, McCoy RG, Lampman MA, et al. Association of Neighborhood Measures of Social Determinants of Health With Breast, Cervical, and Colorectal Cancer Screening Rates in the US Midwest. *JAMA Netw Open*. Mar 2 2020;3(3):e200618. doi:10.1001/jamanetworkopen.2020.0618

100. Berkowitz RL, Bui L, Shen Z, et al. Evaluation of a social determinants of health screening questionnaire and workflow pilot within an adult ambulatory clinic. *BMC Fam Pract*. Dec 24 2021;22(1):256. doi:10.1186/s12875-021-01598-3

101. Stelson E, Mogul M, Harner H, Grisso JA, Frasso R. Social Determinants of Health and What Mothers Say They Need and Want After Release From Jail. *Prev Chronic Dis*. Dec 6 2018;15:E150. doi:10.5888/pcd15.180260

102. Buitron de la Vega P, Losi S, Sprague Martinez L, et al. Implementing an EHR-based Screening and Referral System to Address Social Determinants of Health in Primary Care. *Med Care*. Jun 2019;57 Suppl 6 Suppl 2:S133-s139. doi:10.1097/mlr.0000000000001029

103. Costich MA, Peretz PJ, Davis JA, Stockwell MS, Matiz LA. Impact of a Community Health Worker Program to Support Caregivers of Children With Special Health Care Needs and Address Social Determinants of Health. *Clin Pediatr (Phila)*. Oct 2019;58(11-12):1315-1320. doi:10.1177/0009922819851263

104. Cusack M, Montgomery AE, Hunt-Johnson N, Dichter M, True G. Making Connections: Understanding How Screening, Triage, and Referral Processes Can Promote Veteran Housing Stability. *Soc Work Public Health*. 2019;34(6):483-491. doi:10.1080/19371918.2019.1629143

105. Power-Hays A, Li S, Mensah A, Sobota A. Universal screening for social determinants of health in pediatric sickle cell disease: A quality-improvement initiative. *Pediatr Blood Cancer*. Jan 2020;67(1):e28006. doi:10.1002/pbc.28006

106. Albright DL, Johnson K, Laha-Walsh K, McDaniel J, McIntosh S. Social Determinants of Opioid Use among Patients in Rural Primary Care Settings. *Soc Work Public Health*. Aug 18 2021;36(6):723-731. doi:10.1080/19371918.2021.1939831

107. Rogers CK, Parulekar M, Malik F, Torres CA. A Local Perspective into Electronic Health Record Design, Integration, and Implementation of Screening and Referral for Social Determinants of Health. *Perspect Health Inf Manag*. Spring 2022;19(Spring):1g.

108. Crusan A, Roozen K, Godoy-Henderson C, Zamarripa K, Remache A. Using Community-Based Participatory Research Methods to Inform the Development of Medically Tailored Food Kits for Hispanic/Latine Adults with Hypertension: A Qualitative Study. *Nutrients*. Aug 17 2023;15(16)doi:10.3390/nu15163600

109. Javed Z, Valero-Elizondo J, Maqsood MH, et al. Social determinants of health and obesity: Findings from a national study of US adults. *Obesity (Silver Spring)*. Feb 2022;30(2):491-502. doi:10.1002/oby.23336

110. Abar B, Hong S, Aaserude E, Holub A, DeRienzo V. Access to Care and Depression among Emergency Department Patients. *J Emerg Med*. Jul 2017;53(1):30-37. doi:10.1016/j.jemermed.2016.11.029

111. Sood RK, Bae JY, Sabety A, Chan PY, Heindrichs C. ActionHealthNYC: Effectiveness of a Health Care Access Program for the Uninsured, 2016-2017. *Am J Public Health*. Jul 2021;111(7):1318-1327. doi:10.2105/ajph.2021.306271

112. Ireson E, Burkhardt MC, DeBlasio D, et al. An Assessment of a Socioeconomic Risk Screening Tool for Telemedicine Encounters in Pediatric Primary Care: A Pilot Study. *Clin Pediatr (Phila)*. May 2023;62(4):349-355. doi:10.1177/00099228221128375

113. Woo Baidal JA, Duong N, Goldsmith J, et al. Association of a primary care-based mobile food pantry with child body mass index: A propensity score matched cohort study. *Pediatr Obes*. Jun 2023;18(6):e13023. doi:10.1111/ijpo.13023

114. Inoue K, Watson KE, Kondo N, et al. Association of Intensive Blood Pressure Control and Living Arrangement on Cardiovascular Outcomes by Race: Post Hoc Analysis of SPRINT Randomized Clinical Trial. *JAMA Netw Open*. Mar 1 2022;5(3):e222037. doi:10.1001/jamanetworkopen.2022.2037

115. Martin KJ, Castano C, Geraghty S, et al. Barriers and Facilitators to Prevention and Care of COVID-19 Infection in Cincinnati Latinx Families: a Community-Based Convergent Mixed Methods Study. *J Racial Ethn Health Disparities*. Jun 2023;10(3):1067-1085. doi:10.1007/s40615-022-01294-7

116. Jamerson T, Sylvester R, Jiang Q, et al. Differences in Cardiovascular Disease Risk Factors and Health Behaviors Between Black and Non-Black Students Participating in a School-Based Health Promotion Program. *Am J Health Promot*. Jul 2017;31(4):318-324. doi:10.1177/0890117116674666

117. Wetherill MS, Williams MB, Hartwell ML, et al. Food choice considerations among American Indians living in rural Oklahoma: The THRIVE study. *Appetite*. Sep 1 2018;128:14-20. doi:10.1016/j.appet.2018.05.019

118. Gerber E, Gelberg L, Rotrosen J, Castelblanco D, Mijanovich T, Doran KM. Health-related material needs and substance use among emergency department patients. *Subst Abus*. 2020;41(2):196-202. doi:10.1080/08897077.2019.1635960

119. Steinman L, Parrish A, Mayotte C, et al. Increasing Social Connectedness for Underserved Older Adults Living With Depression: A Pre-Post Evaluation of PEARLS. *Am J Geriatr Psychiatry*. Aug 2021;29(8):828-842. doi:10.1016/j.jagp.2020.10.005

120. Ijadi-Maghsoodi R, Quan M, Horton J, et al. Youth Growing Up in Families Experiencing Parental Substance Use Disorders and Homelessness: A High-Risk Population. *J Child Adolesc Psychopharmacol*. Dec 2019;29(10):773-782. doi:10.1089/cap.2019.0011

121. Guyatt GH, Oxman AD, Kunz R, Vist GE, Falck-Ytter Y, Schünemann HJ. What is “quality of evidence” and why is it important to clinicians? *BMJ*. 2008;336(7651):995-998. doi:10.1136/bmj.39490.551019.BE

122. Henrikson NB, Blasi PR, Dorsey CN, et al. Psychometric and Pragmatic Properties of Social Risk Screening Tools: A Systematic Review. *Am J Prev Med*. Dec 2019;57(6 Suppl 1):S13-s24. doi:10.1016/j.amepre.2019.07.012
