# Supplementary material for: Standardizing social determinants of health data: a proposal for a comprehensive screening tool to address health equity a systematic review
Source: Health Aff Sch. 2024 Nov 14;2(12):qxae151. doi: 10.1093/haschl/qxae151 (PMC11642620; doi:10.1093/haschl/qxae151)
Supplement: qxae151_Supplementary_Data [file qxae151_supplementary_data.zip › SDOH_Appendix_table2.docx]

**Appendix Table 2 . Psychometric Evidence for Social Risk Screening Tools^122^**

|  | Internal consistency | Convergent Validity | Discriminant Validity | Known groups validity | Predictive validity | Concurrent validity | Structural validity | Responsiveness | Norms | Total |
| --- | --- | --- | --- | --- | --- | --- | --- | --- | --- | --- |
| WE CARE-BMC THRIVE | 0 | 0 | 0 | 0 | 0 | 0 | 0 | 0 | 2 | 2 |
| The Health Leads screening toolkit | 0 | 0 | 0 | 0 | 0 | 0 | 0 | 0 | 0 | 4 |
| PRAPARE tool | 0 | 4 | 0 | 0 | -1 | 0 | 0 | 0 | 2 | 5 |
| The Accountable Health Communities-HRSN tool | 0 | 0 | 0 | 0 | 0 | 0 | 0 | 0 | 0 | 0 |
| CDC Environmental justice index (EJI) | NA | NA | NA | NA | NA | NA | NA | NA | NA | NA |
| EPIC SDOH tool | NA | NA | NA | NA | NA | NA | NA | NA | NA | NA |

Note: Scores within each category range from ‒1 (poor) to 4 (excellent). The minimum total score is ‒9, the maximum total score is 36. NA- Not available
