# Supplementary material for: Standardizing social determinants of health data: a proposal for a comprehensive screening tool to address health equity a systematic review
Source: Health Aff Sch. 2024 Nov 14;2(12):qxae151. doi: 10.1093/haschl/qxae151 (PMC11642620; doi:10.1093/haschl/qxae151)
Supplement: qxae151_Supplementary_Data [file qxae151_supplementary_data.zip › SDOH_Appendix_Table3.docx]

**Appendix Table 3. Pragmatic Evidence for Social Risk Screening Tools^122^**

|  | **Cost** | **Accessible language** | **Ease of Training** | **Ease of Interpretation** | **Brief** | **Total Score** |
| --- | --- | --- | --- | --- | --- | --- |
| **WE CARE-BMC THRIVE** | 4 | 4 | 4 | 1 | 4 | 17 |
| **The Health Leads screening toolkit** | 3 | 4 | 2 | 1 | 4 | 13 |
| **PRAPARE tool** | 4 | 4 | 2 | 1 | 3 | 14 |
| **The Accountable Health Communities-HRSN tool** | 4 | 3 | 0 | 0 | 3 | 10 |
| **CDC Environmental justice index (EJI)** | NA | NA | NA | NA | NA | NA |
| **EPIC SDOH tool** | NA | NA | NA | NA | NA | NA |

Note: Scores within each category range from ‒1(poor) to 4 (excellent). The minimum total score is‒5, the maximum total score is 20. NA- Not available
