# Supplementary material for: Standardizing social determinants of health data: a proposal for a comprehensive screening tool to address health equity a systematic review
Source: Health Aff Sch. 2024 Nov 14;2(12):qxae151. doi: 10.1093/haschl/qxae151 (PMC11642620; doi:10.1093/haschl/qxae151)
Supplement: qxae151_Supplementary_Data [file qxae151_supplementary_data.zip › SDOH_Appendix_Table4.docx]

**Appendix Table 4 . References for the SDOH domain screening survey of the proposed tool**

| **Tool SDOH Domain** | **Item #** | **Question** | **Reference** |
| --- | --- | --- | --- |
| Minority Status | 1,2 | What is your race?  What is your ancestry or ethnic origin? | United States Census Bureau. The American Community Survey. American Community Survey. Published 2022. Accessed May 30, 2023. www2.census.gov/programs-surveys/acs/methodology/questionnaires/2022/quest22.pdf |
| Socio-economic factors | 3,4,5,10 | What was your total household income during the PAST 12 MONTHS?  What is your highest degree or level of school you have COMPLETED?  Are you currently employed?  If “yes” to the above, please answer item 5a) below. If “no” to the above, please skip to item    5a. a) Full-time? b) Part-time c) Variable  5b). When did you last work, even for a few days?  Are you CURRENTLY covered by any of the following types of health insurance or health coverage plans?    10a.Insurance through a current or former employer or union (of this person or another family member)?  10b. Insurance purchased directly from an insurance company (by this person or another family member)?  10c. Medicare, for people 65 and older, or people with certain disabilities?  10d. Medicaid, Medical Assistance, or any kind of government-assistance plan for those with low incomes or a disability?  10e. TRICARE or other military health care?  10f. VA (enrolled for VA health care)?  10g. Indian Health Service?  10h. Any other type of health insurance or health coverage plan1 – Specify _________________ | United States Census Bureau. The American Community Survey. American Community Survey. Published 2022. Accessed May 30, 2023.  www2.census.gov/programs-surveys/acs/methodology/questionnaires/2022/quest22.pdf  National Center for Health Statistics. National health Interview Survey.  <https://ftp.cdc.gov/pub/health_Statistics/nchs/Survey_Questionnaires/NHIS/2018/english/qadult.pdf> |
| Financial resource strain | 6c | What is the monthly mortgage, loan or rent for this house, apartment, or mobile home? | United States Census Bureau. The American Community Survey. American Community Survey. Published 2022. Accessed May 30, 2023. www2.census.gov/programs-surveys/acs/methodology/questionnaires/2022/quest22.pdf |
| Household Type | 6,7 | 6a.What is your housing situation today?  6b.Is this house, apartment, or mobile home?  7.Which best describes your building? | United States Census Bureau. The American Community Survey. American Community Survey. Published 2022. Accessed May 30, 2023. www2.census.gov/programs-surveys/acs/methodology/questionnaires/2022/quest22.pdf |
| Household characteristics | 8,14 | 8a. How many people are living or staying at your home address?  8b. How many separate bedrooms are in this house, apartment or mobile home?  14a. What is your marital status?  14b. Are you a single parent? (Male/female householder, no spouse or partner present, with own children under 18 years) | United States Census Bureau. The American Community Survey. American Community Survey. Published 2022. Accessed May 30, 2023. www2.census.gov/programs-surveys/acs/methodology/questionnaires/2022/quest22.pdf |
| Environment Burden | 9 | How long have you been at your current residence?  What is the zip code of your longest-lived residence? | Ompad, D. C., Galea, S., Caiaffa, W. T., & Vlahov, D. (2007). Social determinants of the health of urban populations: methodologic considerations. Journal of urban health : bulletin of the New York Academy of Medicine, 84(3 Suppl), i42–i53. <https://doi.org/10.1007/s11524-007-9168-4> |
| Access to Phone and Internet | 11 | At this house, apartment, or mobile home –do you or any member of this household have access to the Internet | United States Census Bureau. The American Community Survey. American Community Survey. Published 2022. Accessed May 30, 2023. www2.census.gov/programs-surveys/acs/methodology/questionnaires/2022/quest22.pdf |
| Disability | 12 | 12a. Are you deaf or do you have serious difficulty hearing?  12b. Are you blind or do you have serious difficulty seeing, even when wearing glasses?  12c. Because of a physical, mental, or emotional condition, do you have serious difficulty concentrating, remembering, or making decisions?  12d. Do you have serious difficulty walking or climbing stairs?  12e. Do you have difficulty dressing or bathing?  12f. Because of a physical, mental, or emotional condition, do you have difficulty doing errands alone such as visiting a doctor's office or shopping? | 2021 BRFSS Questionnaire. Behavioral Risk Factor Surveillance System. <https://www.cdc.gov/brfss/questionnaires/pdf-ques/2021-BRFSS-Questionnaire-1-19-2022-508.pdf>  Centers for Medicare & Medicaid Services. The Accountable Health Communities Health-Related Social Needs Screening Tool. Centers for Medicare & Medicaid Services. Published 2023. Accessed May 30, 2023. [https://innovation.cms.gov/files/worksheets/ahcm-screeningtool.pdf](about:blank) |
| Language | 13 | How well do you speak English? | United States Census Bureau. The American Community Survey. American Community Survey. Published 2022. Accessed May 30, 2023. www2.census.gov/programs-surveys/acs/methodology/questionnaires/2022/quest22.pdf |
| Transportation | 15. | 15a. How did you usually get to work LAST WEEK? Mark (X) ONE box for the method of transportation used for most of the distance?  15b. In the past 12 months, has lack of reliable transportation kept you from medical appointments, meetings, work, or from getting things needed for daily living? | United States Census Bureau. The American Community Survey. American Community Survey. Published 2022. Accessed May 30, 2023. www2.census.gov/programs-surveys/acs/methodology/questionnaires/2022/quest22.pdf |
| Access to food, utilities, and healthcare | 16 | In the past year, have you or any family members you live with been unable to get any of the following when it was really needed? Check all that apply.  -Food  -Healthy food (fresh fruits and vegetables)  -Utilities  -Medicines or Healthcare (Medical, Dental, Mental Health, Vision)  -Phone  -I choose not to answer this  If you responded “yes” to any of the above, please answer the following questions:  a) DURING THE PAST 12 MONTHS, please indicate if you or anyone in the family had problems paying or were unable to pay any medical bills?  b) DURING THE PAST 12 MONTHS, has medical care been delayed because of worry about the cost?  c) How worried are you right now about not being able to pay your rent, mortgage, or other housing costs?  d) How worried are you right now about not being able to pay your normal monthly bills? | National Association of Community Health Centers. Protocol for Responding to and Assessing Patient Assets, Risks, and Experiences. National Association of Community Health Centers. Published 2020. Accessed May 30, 2023. [https://prapare.org/](about:blank)  National Center for Health Statistics. National health Interview Survey.  https://ftp.cdc.gov/pub/health_Statistics/nchs/Survey_Questionnaires/NHIS/2018/english/qadult.pdf |
| Social Connection | 17 | How often do you see or talk to people that you care about and feel close to? (For  example: talking to friends on the phone, visiting friends or family, going to church or club meetings | National Association of Community Health Centers. Protocol for Responding to and Assessing Patient Assets, Risks, and Experiences. National Association of Community Health Centers. Published 2020. Accessed May 30, 2023. [https://prapare.org/](about:blank) |
|  | 18 | How much do you agree or disagree with the following statements about your neighborhood? Would you say… There are people I can count on in this neighborhood? | National Center for Health Statistics. National health Interview Survey.  https://ftp.cdc.gov/pub/health_Statistics/nchs/Survey_Questionnaires/NHIS/2018/english/qadult.pdf |
| Stress | 19a and 19b | 19a.Over the last 2 weeks, how often have you been bothered by the following problems?  1. Little interest or pleasure in doing things.  2. Feeling down, depressed or hopeless  19b. How often does your partner:  A. Physically hurt you?  B. Insult you or talk down to you?  C. Threaten you with harm?  D. Scream or curse at you? | 19a. Kroenke, K., Spitzer, R. L., & Williams, J. B. (2003). The Patient Health Questionnaire-2: validity of a two-item depression screener. Medical care, 41(11), 1284–1292. <https://doi.org/10.1097/01.MLR.0000093487.78664.3C>  19b.Rabin, R. F., Jennings, J. M., Campbell, J. C., & Bair-Merritt, M. H. (2009). Intimate partner violence screening tools: a systematic review. American journal of preventive medicine, 36(5), 439–445.e4. <https://doi.org/10.1016/j.amepre.2009.01.024> |
| Health Behaviors | 20 | In the last 30 days, other than the activities you did for work, on average, how many days per week did you engage in moderate exercise (like walking fast, running, jogging, dancing, swimming, biking, or other similar activities)?  On average, how many minutes did you usually spend exercising at this level on one of those days? | Centers for Medicare & Medicaid Services. The Accountable Health Communities Health-Related Social Needs Screening Tool. Centers for Medicare & Medicaid Services. Published 2023. Accessed May 30, 2023. [https://innovation.cms.gov/files/worksheets/ahcm-screeningtool.pdf](about:blank) |
