# Supplementary material for: Standardizing social determinants of health data: a proposal for a comprehensive screening tool to address health equity a systematic review
Source: Health Aff Sch. 2024 Nov 14;2(12):qxae151. doi: 10.1093/haschl/qxae151 (PMC11642620; doi:10.1093/haschl/qxae151)
Supplement: qxae151_Supplementary_Data [file qxae151_supplementary_data.zip › Supplemental file.docx]

**Supplemental Appendix**

**PubMed Search**

**Search strategy:**  (((((((((("Social Determinant"[All Fields] OR "Socioeconomic Factors"[All Fields] OR "Patient Advocacy"[All Fields] OR "Social Welfare"[All Fields] OR "Educational Status"[All Fields] OR "Employment"[All Fields] OR "unemployment"[All Fields] OR "Food Supply"[All Fields] OR "Food Assistance"[All Fields] OR “Food Insecurity” [All Fields] OR "Housing"[All Fields] OR "Homeless"[All Fields] OR "Domestic Violence"[All Fields] OR "Public Assistance"[All Fields] OR "childcare"[All Fields] OR "Social Isolation"[All Fields] OR "Transportation"[All Fields] OR (Medical-Legal[All Fields] AND Partnership[All Fields])) AND ("Primary Health Care"[All Fields] OR "Patient-Centered Care"[All Fields] OR "Patient Care Team"[All Fields] OR "Health Services"[All Fields] OR "Delivery of Health Care"[All Fields] OR "Community Health Services"[All Fields])) AND ("Intervention"[All Fields] OR "Organizational Case Studies"[All Fields] OR "Program Development"[All Fields] OR "Referral and Consultation"[All Fields] OR "Pilot Projects"[All Fields] OR "Community Health Worker"[All Fields] OR "Patient Navigator"[All Fields] OR "Needs Assessment"[All Fields])) NOT ("africa"[MeSH Terms] OR "africa"[All Fields])) NOT ("south america"[MeSH Terms] OR ("south"[All Fields] AND "america"[All Fields]) OR "south america"[All Fields])) NOT ("asia"[MeSH Terms] OR "asia"[All Fields])) NOT ("australia"[MeSH Terms] OR "australia"[All Fields])) NOT ("latin america"[MeSH Terms] OR ("latin"[All Fields] AND "america"[All Fields]) OR "latin america"[All Fields])) NOT ("south africa"[MeSH Terms] OR ("south"[All Fields] AND "africa"[All Fields]) OR "south africa"[All Fields]))

**Rationale for SDoH screening**

Evidence from randomized trials, pilot projects, and epidemiological studies demonstrates that interventions across SDOH domains like housing, food, transportation, income, and education can improve health outcomes and lower costs.^8^ Appendix Table 1 highlights the importance of taking action on social determinants of health (SDOH) screening in clinical settings.^8^ While many studies have examined SDOH screening alone, this table focuses on studies of interventions that go beyond screening to address patients' identified social needs.^8^ The table shows that studies of SDOH interventions, compared to screening alone, are more likely to examine impacts on health outcomes and healthcare utilization. ^8^This suggests that taking action on SDOH screening by connecting patients to resources can lead to measurable improvements in health and reductions in inappropriate healthcare utilization.^8^ As clinical sites consider implementing SDOH screening, they should ensure adequate capacity to address identified needs through internal resources, partnerships, or referrals. Simply screening without the ability to act limits the clinical and population health impact.^8^ By planning for follow-up on identified needs during the design phase, health systems can maximize the benefits of SDOH screening for patients and communities.

**Appendix Table 1 .** Summary of SDoH studies, interventions and outcomes.^8^

|  | **Author, year** | **Target social need** | **Intervention** | **Clinical setting** | **Study design, sample type, and size** | **Summary of changes in outcomes post intervention** |
| --- | --- | --- | --- | --- | --- | --- |
| 1. | Tomita and Herman, 2015^45^ | Homelessness | Critical time intervention (CTI), time-limited case management model focused on building and strengthening community support systems during critical transition periods. | Inpatient care | **Study design:** RCT **Sample:** 150 severely mentally ill, previously homeless adults discharged from inpatient psychiatric care (77  intervention, 73 control) | **Findings:** No significant differences in continuity of care process measures (perceived ease of access to care process, stability, or severity of instability of patient- provider relationship.  **SDoH:** None reported  **Provider:** None reported.  **Health:** None reported  **Cost/Utilization:** None reported |
| 2. | Garg et al., 2015^46^ | Multiple Social Determinants of Health (education, employment, food security, household heat, and housing.) | Well Child Care, Evaluation, Community Resources, Advocacy, Referral, Education (WE CARE), social needs screening and referral tool | Pediatric primary care clinic | **Study design:** Cluster RCT in eight urban Community Health Centers.  **Sample:** 336 mothers of healthy infants (168  intervention, 168 control). | **Findings:** Increased change in number of social referrals; Increased change enrollment in community resources.  **SDoH:** Increased change in childcare enrollment, employment, receipt of fuel assistance, and stable housing.  **Provider:** None reported  **Health:** None reported  **Cost/Utilization:** None reported |
| 3 | Kangovi, et al., 2014^47^ | Socio-economic status | Individualized Management for Patient Centered Targets (IMPaCT), CHWs work with hospitalized patients at admission and post-discharge to create individualize action plans related to socioeconomic and behavior barriers to care. | Urban hospital (inpatient care) | **Study design:** RCT **Sample:** 446 low-income, uninsured or Medicaid general medical patients were randomized into IMPaCT intervention (n=222) or usual care (n=224). | **Findings:** CHWs helped patients achieve half of action plan goals and were present at less than half of discharges and PCP follow up visits; Increased open-ended feedback from participants in intervention arm.  **SDoH:** None reported.  **Provider:** None reported  **Health:** Increased change in mental health, patient activation and quality of discharge communication; no significant change in physical health, patient satisfaction with medical care, or medication adherence.  **Cost/Utilization:** Increased change in post-discharge primary care follow-up; reduction in multiple hospital readmissions. |
| 4 | Mendelson et al, 2011^48^ | Education/ Literacy | Video Interaction Project (VIP) and Building Blocks (BB), primary care interventions that build on ROR by beginning earlier (0-3 years), and by providing materials and strategies for enhancing verbal interactions. VIP includes in-person sessions with child development specialist utilizing taped mother-child interactions. BB is delivered through newsletters and learning materials sent home monthly. | Pediatric primary care | **Study design:** Single- blind 3-way RCT **Sample:** 675 mother- infant dyads were randomized into VIP intervention (n=225), BB intervention (n=225) or control (225). | **Findings:** None reported  **SDoH:** Increased change in parent-child literacy-building activities and child language development were observed in both VIP and BB interventions.  **Provider:** None reported  **Health:** None reported  **Cost/Utilization:** None reported |
| 5 | Klevens et al., 2012^49^ | IPV | Clinic-based computer IPV screening and resource referral | Primary care | **Study design:** RCT  **Sample:** 2,708 women seen in 10 primary healthcare centers were randomized into three intervention groups: (1) partner violence screen (using the Partner Violence Screen instrument) plus a list of local partner violence resources if screening was positive (n=909); (2) partner violence resource list only without screen (n=893); and (3) no- screen, no-partner violence list control group (n=898) | **Findings:** No significant impact on linkages to community-based resources.  **SDoH:** No significant impact on IPV recurrence.  **Provider:** None reported  **Health:** No significant impact on quality of life  **Cost/Utilization:** No significant impact on healthcare utilization |
| 6 | Silverstein et al., 2004^50^ | Socio-economic status | Clinic-based referral system to Head Start | Pediatric primary care | **Study design:** RCT **Sample:** 246 Head Start eligible children (123  intervention, 123 control). | **Findings:** Increased change in child Head Start attendance.  **SDoH:** None reported **Provider:** None reported **Health:** None reported  **Cost/Utilization:** None reported |
| 7 | Herman et al., 2011^51^ | Homelessness | Critical time intervention (CTI), time-limited case management model focused on building and strengthening community support systems during critical transition periods. | Inpatient care | **Study design:** RCT **Sample:** 150 severely mentally ill, previously homeless adults discharged from inpatient psychiatric care (77  intervention, 73 control) | **Findings:** None reported  **SDoH:** Reduction in homelessness.  **Provider:** None reported  **Health:** None reported  **Cost/Utilization:** None reported |
| 8 | Tomita and Herman, 2012^52^ | Homelessness | Critical time intervention (CTI), time-limited case management model focused on building and strengthening community support systems during critical transition periods. | Inpatient care | **Study design:** RCT **Sample:** Of the 150 participants, 73 participants (49%) were randomly assigned to receive usual services only while 77 participants (51%) were assigned to receive CTI in addition to usual services. | **Findings:** None reported  **SDoH:** None reported.  **Provider:** None reported  **Health:** None reported  **Cost/Utilization:** Reduction in psychiatric re- hospitalizations. |
| 9 | Garg et al., 2007^53^ | Multiple SDoH (parental depression, substance  use, intimate-partner violence, parental abuse, social  supports, housing, single parent, less than high school  education, and unemployed) | WE CARE, social needs screening and referral tool | Pediatric primary care clinic | **Study design:** RCT **Sample:** 200 parents of children age 2 months to 10 years (100 intervention,  100 control) and 45  residents (24 intervention,  21 control). | **Findings:** Increased change in number of psychosocial needs discussed; Increased change in referrals provided; and Increased change in contact of community resource.  **SDoH:** None reported  **Provider:** Residents reported survey did not slow visit.  **Health:** None reported  **Cost/Utilization:** None reported |
| 10 | Gottlieb et al., 2016^54^ | Multiple SDoH (housing stability and habitability, food and income security, child care and transportation needs, employment, legal concerns, medical insurance and other public benefits enrollment, and concerns about any adult household member’s mental health.) | Volunteer patient navigators provide clinic-based social needs screening and connect families to resources. | Pediatric primary and urgent care clinics | **Study design:** RCT  **Sample:** 1,809 families  with children (872  intervention, 937 control). | **Findings:** Increased identification of social needs; most frequently reported needs related to money for food and utilities, employment, and housing.  **SDoH:** Decreased number of social needs.  **Provider:** None reported  **Health:** Increased change in parent reported child health (d=0.16).^a^  **Cost/Utilization:** None reported |
| 11 | Waitzkin, 2011^55^ | Multiple SDoH (underemployment, inadequate housing, food insecurity, and violence). | Clinic-based community health worker (*promotora)* to address contextual sources of depression | Community Health Centers (CHCs) | **Study design:** Cluster RCT in two CHCs and ethnographic assessment **Sample:** 120 patients with depression were randomized to enhanced care plus the CHW contextual intervention, or to enhanced care alone. | **Findings:** Increased response to intervention by patients, *promotoras*, providers, clinic staff, and administrators. **SDoH:** No significant change in contextual sources of depression.  **Provider:** None reported  **Health:** No significant change in depression.  **Cost/Utilization:** None reported |
| 12 | Bronstein et al., 2015^56^ | Socio-economic status | Social work-led care coordination designed to identify and alleviate barriers to patients remaining at home post-hospital discharge. | Nonprofit regional hospital | **Study design:** RCT  **Sample:** 89 adults, aged  ≥50 years with moderate to high risk of 30-day hospital readmission post- discharge were randomized into social work intervention (n=45) or usual care (n=44). | **Findings:** Increased patient response to intervention.  **SDoH:** None reported **Provider:** None reported **Health:** None reported  **Cost/Utilization:** Increased change in post-discharge risk for 30- day hospital readmission. |
| 13 | Berkowitz et al., 2016^57^ | Multiple SDoH (Health needs,  Employment,  Financial,  Food,  Housing,  Legal,  Transportation  Utilities) | Health Leads (formally Project HEALTH), social needs screening and referral physicians “prescribe” social resource and health leads volunteers assist patients by providing relevant community  resources. | Adult primary care clinics | **Study design:** Difference in difference evaluation **Sample:** 5,125 adult patients screened between October 1, 2013 and April  30, 2015. (1,774 allocated to health leads; 3,351 comparison) | **Findings:** Increased identification of social needs; Increased change in connections to and enrollment in community programs. **SDoH:** None reported  **Provider:** None reported  **Health:** Increased change in improvement of systolic and diastolic blood pressure; Increased change in low-density lipoprotein cholesterol (LDL-C) level. No change in HbA1c levels observed.  **Cost/Utilization:** None reported |
| 14 | Sege et al.,  2015^58^ | Legal needs | Project Developmental Understanding and Legal Collaboration for Everyone (DULCE), Family Specialists conduct needs assessments and works directly with families to provide connections to resources. | Pediatric primary care | **Study design:** RCT **Sample:** 330 families of newborns <10 weeks (167  intervention; 163 control) | **Findings:** Increased increase in access to attainable concrete supports (e.g., food, public benefits, discounted telephone service); no differences observed in access to supports judged unobtainable during study period (e.g., housing). Family specialists met with intervention parents an average of 14 times for a total of 5 hours.  **SDoH:** None reported  **Provider:** None reported  **Health:** Increased increase in preventive care (infants in intervention significantly more likely to receive routine immunizations before 7 months (d=0.42); before 8 months (d=0.45); or ever (d=0.50).  **Cost/Utilization:** Decreased emergency department visits by age 6 months. |
| 15 | Feigelman et al., 2011^59^ | Child Maltreatment | Safe Environment for Every Kid (SEEK), providers identify and address psychosocial problems associated with child maltreatment. | Pediatric primary care | **Study design:** RCT **Sample:** 95 residents (45 usual care, 45 SEEK). | **Findings:** ; Increased change in patient satisfaction with child’s doctor.  **SDoH:** None reported  **Provider:** Increased change in resident comfort and screening practices for psychosocial risk factors.  **Health:** None reported  **Cost/Utilization:** None reported |
| 16 | Morales et al., 2016^60^ | Food Insecurity | Food for Families identifies food insecure families during clinic visit and connects them with food resources (e.g., SNAP, WIC, and  food pantries). | Community health center obstetric clinic | **Study design:**  Retrospective propensity score-matched analysis. **Sample:** Adult women aged ≥18 years who visited clinic from June 1, 2013 through June 1,  2015. 145 of 1,295 seen in clinic were referred to Food for Families. | **Findings:** None reported  **SDoH:** None reported **Provider:** None reported  **Health:** Enrollment in program associated with better blood pressure trends; no trends in blood glucose levels were observed.  **Cost/Utilization:** None reported |
| 17 | Dubowitz et al., 2011^61^ | Child Maltreatment | Safe Environment for Every Kid (SEEK), providers identify and address psychosocial problems associated with child maltreatment. | Pediatric primary care | **Study design:** Cluster RCT in 18 private pediatric medical practices.  **Sample:** 102 healthcare  professionals (56 from SEEK model practices, 46  from control practices) | **Findings:** Increased change in screening and intervention activities around maternal factors associated with child maltreatment.  **SDoH:** None reported  **Provider:** Increased change in comfort level and perceived competence in addressing maternal factors associated with child maltreatment.  **Health:** None reported  **Cost/Utilization:** None reported |
| 18 | Dubowitz et al., 2012^62^ | Child Maltreatment | Safe Environment for Every Kid (SEEK), providers identify and address psychosocial problems associated with child maltreatment. | Pediatric primary care | **Study design:** Cluster RCT in 18 private pediatric medical practices.  **Sample:** 1,119 mothers of children aged 0 to 5 years (595 from SEEK model practices, 524 from control practices). | **Findings:** No differences in time spent on the intervention or time spent discussing psychosocial concerns in SEEK and control sites.  **SDoH:** Reduced child maltreatment.  **Provider:** None reported **Health:** None reported **Cost/Utilization:** None reported |
| 19 | Krieger et al., 2005^63^ | Housing/ Habitability | Seattle-King County Healthy Homes Project, community health worker (CHW) provides home assessments, individualized action plans, education, materials, and advocacy to improve housing  conditions. | Urgent care, hospitals, Community Health Centers | **Study design:** RCT **Sample:** 274 low-income children with asthma aged 4-12 years (138 high  intensity intervention, 136 low intensity intervention). | **Findings:** CHWs made a mean number of 7 visits lasting 1 hour.  **SDoH:** Increased actions to reduce triggers.  **Provider:** None reported  **Health:** Increased change in caregiver quality of life scores and reduction in asthma-related urgent services use (d= -0.43). No significant decline in asthma symptom days.  **Cost/Utilization:** Reported projected 4-year net savings. |
| 20 | Hassan et al.,  2015^64^ | Financial security, Healthcare access | HelpSteps (The Online Advocate), self-administered web-based health- related social domains screening and referral tool | Urban hospital- based adolescent and young adult clinic | **Study design:** Prospective descriptive analysis.  **Sample:** 401 youth and young adults aged 15-25 | **Findings:** Increased identification of social needs. Income security, nutrition and fitness, and healthcare access most frequently reported as priority domains. Almost half of youth reported contacting referral agency.  **SDoH:** Increased change in complete or partial resolution of priority problem.  **Provider:** None reported  **Health:** None reported  **Cost/Utilization:** None reported |
| 21 | Haas et al.,  2015^65^ | Socio-economic status | (1) telephone-based motivational counseling, (2) free nicotine replacement therapy (NRT) for 6 weeks, (3) access to community-based referrals to address socio-contextual mediators of tobacco use, and (4) integration of all these components into their normal health care through the EHR system. | Primary care practices | **Study design:** Subgroup analysis of intervention arm of RCT  **Sample:** 274 adult smokers | **Findings:** Less than half of intervention participants requested community referrals; use of referrals not widely reported.  **SDoH:** None reported  **Provider:** None reported  **Health:** Quitting smoking status did not differ by request for community referral. Participants who reported use of referral were more likely quit smoking than those who did not.  **Cost/Utilization:** None reported |
| 22 | Becker et al.,  2004^66^ | Socio-economic status, interpersonal violence | Caught in the Crossfire, hospital- based intensive peer advocate case management program. Peer advocates work with violently injured youth to identify and address social needs. | Urban county hospital | **Study design:** Retrospective case-control study.  **Sample:** 112 hospitalized violently injured youth aged 12-20 years.  Treatment (n=43) and control (n=69) youth were matched by age and injury  severity. | **Findings:** None reported  **SDoH:** Reduction in youth involvement in criminal justice system.  **Provider:** None reported  **Health:** No significant change in mortality.  **Cost/Utilization:** No significant change in rates of re- hospitalization for re-injury. |
| 23 | Dicker et al.,  2009^67^ | Multiple SDoH (Education status; School fighting; Employment status; Attrition rate from program; Social circle; Attrition from mental health services; Substance abuse  Fulfillment of other needs) | The Wraparound Project, hospital- based case management program targeting violently injured youth.  Culturally competent case managers create risk reduction plan and shepherd clients through community resources. | Level I trauma center | **Study design:** Quality Improvement program evaluation.  **Sample :** 45 high risk youth. | **Findings:** Case managers successfully screened target population and spent significant amount of time with high-risk clients. No correlation was found between number of needs and time spent with case manager.  **SDoH:** Reduction in social needs (Six of seven major needs were resolved at least half of the time.)  **Provider:** None reported  **Health:** None reported  **Cost/Utilization:** None reported |
| 24 | Juillard et al.,  2016^68^ | Multiple SDoH (Employment, Housing and IPV) | The Wraparound Project, hospital- based case management program targeting violently injured youth. Culturally competent case managers create risk reduction plan and shepherd clients through community resources. | Level I trauma center | **Study design:** Longitudinal observational analysis of prospectively collected program and trauma registry data from 2005- 2014.  **Sample:** 466 violently injured youth and adults aged 10-35 years. | **Findings:** Increased identification social needs; mental health services, victim of crime compensation, employment ,, housing most commonly identified needs.  **SDoH:** Successfully met victim of crime compensation and visa needs. Low success in meeting employment needs and attaining driver’s licenses.  **Provider:** None reported **Health:** None reported **Cost/Utilization:** None reported |
| 25 | Garg et al., 2012^69^ | Multiple SDoH (Health needs,  Employment,  Financial,  Food,  Housing,  Legal, child care,  Transportation  Utilities) | HealthLeads (formally Project HEALTH), social needs screening and referral physicians “prescribe” social resource and health leads volunteers assist patients by providing relevant community resources. | Pediatric clinics | **Study design:** Prospective cohort study  **Sample:** Data collected on low-income families accessing the program between July 2008 and January 2011 | **Findings:** Increased identification of social needs; Increased change in connections to and enrollment in community programs.  **SDoH:** None reported  **Provider:** None reported **Health:** None reported **Cost/Utilization:** None reported |
| 26 | Paris, 2008^70^ | Multiple SDoH(Education, Social isolation, Poverty) | Visiting Moms, para-professional bilingual and bicultural home visitors provide support and referrals to resources for  immigrant mothers. | Community Health Center | **Study design:** Qualitative interviews  **Sample:** 14 Latina immigrants participating in the program. | **Findings:** Patients reported believing the program was helpful in providing emotional support, case management/advocacy, translation, education, and friendship.  **SDoH:** None reported **Provider:** None reported **Health:** None reported.  **Cost/Utilization:** None reported |
| 27 | Garg et al.,  2010^71^ | Multiple SDoH (Health needs,  Employment,  Financial,  Food,  Housing,  Legal,  Transportation  Utilities) | HealthLeads (formally Project HEALTH), social needs screening and referral physicians “prescribe” social resource and health leads volunteers assist patients by providing relevant community  resources. | Pediatric clinics | **Study design:** Longitudinal cohort pilot  **Sample:** 59 parents of children attending a scheduled clinic visit. | **Findings:** Increased change in identification of social needs;  Increased change in connections to and enrollment in community programs.  **SDoH:** None reported **Provider:** None reported **Health:** None reported  **Cost/Utilization:** None reported |
| 28 | Berkowitz et al., 2016^57^ | Multiple SDoH (Health needs,  Employment,  Financial,  Food,  Housing,  Legal,  Transportation  Utilities) | HealthLeads (formally Project HEALTH), social needs screening and referral physicians “prescribe” social resource and health leads volunteers assist patients by providing relevant community  resources. | Hospital-based primary care clinics | **Study design:** Cross- sectional descriptive study **Sample:** 3,166 patients  (461 reported unmet needs; 2,750 no reported needs) screened in clinic between October 1, 2013  and April 30, 2014. | **Findings:** Increased identification of social needs; successfully closed cases and connected patients to resources.  Affording health care and food commonly reported needs.  **SDoH:** None reported **Provider:** None reported **Health:** None reported  **Cost/Utilization:** None reported |
| 29 | Rodabaugh et al., 2010^72^ | Legal needs | The Legal Services Program: a Medical- Legal Partnership incorporated as part of a comprehensive palliative care model addresses patient unmet social and  material needs. | Comprehensive cancer center | **Study design:** Descriptive analysis of retrospective cohort.  **Sample:** Referrals made to the Legal Services Program (n=247) from April 2004 through  December 2007. | **Findings:** Increased change in number of legal referrals. **SDoH:** Increased change in number of resolved legal issues. **Provider:** None reported  **Health:** None reported  **Cost/Utilization:** Increased change in reimbursement from overturned benefits denials. |

| 30 | Ryan et al.,  2012^73^ | Legal needs | Tucson Family Advocacy Program (TFAP), Medical- Legal Partnership providing free legal services to referred  low-income patients. | Family and Community Medicine clinic | **Study design:** Pre/post prospective cohort study. **Sample:** 104 adult patients or parents of minor patients referred to TFAP for legal services. | **Findings:** None reported **SDoH:** None reported **Provider:** None reported  **Health:** Increased change in overall wellbeing and stress reduction.  **Cost/Utilization:** None reported |
| --- | --- | --- | --- | --- | --- | --- |
| 31 | Weintraub et al., 2010^74^ | Legal needs | Peninsula Family Advocacy Program (FAP), Medical- Legal Partnership serving clinic and hospital-based pediatric populations. | Pediatric primary care | **Study design:** Pre/post prospective cohort study. **Sample:** 102 families receiving services from FAP. | **Findings:** Increased change in families’ use of food and income supports. Majority of patients found program helpful. **SDoH:** Increased change in resolved legal issues.  **Provider:** None reported  **Health:** Increased change in child health and wellbeing. Reduction in avoided health care secondary to lack of health insurance. No significant change in immunization status.  **Cost/Utilization:** No significant change in acute care or emergency department and well child visits |
| 32 | O’Sullivan et al, 2012^75^ | Legal needs | Legal Health, Medical-Legal  Partnership providing hospital-based free weekly legal clinic. | Asthma clinic | **Study design:** Pre/post retrospective cohort study. **Sample:** 12 adult patients with poorly controlled asthma and domestic allergen exposure received legal assistance through LegalHealth. | **Findings:** None reported  **SDoH:** Increased change in housing conditions.  **Provider:** None reported  **Health:** Increased change in asthma control.  **Cost/Utilization:** Reduction in emergency department  visits and hospital admissions. |
| 33 | Cohen et al., 2016^76^ | Food Insecurity | Waiting room explanation and program materials of Double Up Food Bucks (DUFB), a statewide healthy food incentive that matches Supplemental Nutrition Assistance Program (SNAP) funds spent at  farmers markets. | Urban primary care | **Study design:** Longitudinal, repeated measures, quasi- experimental trial **Sample:** 177 SNAP enrolled, adults. | **Findings:** Increased change in use of DUFB.  **SDoH:** None reported  **Provider:** None reported  **Health:** Increased change in daily fruit and vegetable consumption.  **Cost/Utilization:** None reported |
| 34 | Beck et al., 2014^77^ | Food Insecurity | Keeping Infants Nourished and Developing (KIND), a collaborative intervention between pediatric clinics.  Families are screened for food insecurity in clinic and provided with supplemental formula, educational brochures, and referrals. | Pediatric primary care | **Study design:** Quasi- experimental prospective cohort. Analysis of data collected between June 1, 2011 and May 31, 2013.  **Sample:** 5,071 infants seen for well-child care (1,042 received KIND,4,029 no KIND). | **Findings:** Increased change in numbers of children connected to additional clinic resources, including social work and MLP.  **SDoH:** None reported  **Provider:** None reported  **Health:** Increased change in completion of lead test and developmental screen. No impact observed on weight- for-length percentile at 9 months, numeric lead level, or ASQ (Ages and Stages questionnaire developmental screen).  **Cost/Utilization:** Increased change in receipt of preventive services. |
| 35 | Needleman et al., 2005^78^ | Education / Literacy | Reach Out and Read (ROR), families with babies and young children receive books and literacy guidance at well- child visits. | Primary care clinical sites | **Study design:** Pre/post comparison of separate convenience samples **Sample:** Parents of children aged 6-72 months seeking routine health care at 19 clinical sites in 10 states were surveyed pre- intervention (n=730) and post-intervention (n=930) implementation. | **Findings:** None reported  **SDoH:** Increased change in parent-child home reading activities.  **Provider:** None reported **Health:** None reported **Cost/Utilization:** None reported |
| 36 | Mendelsohn et al, 2001^79^ | Education / Literacy | Reach Out and Read (ROR), families with babies and young children receive books and literacy guidance at well- child visits. | Urban pediatric clinics | **Study design:** Quasi- experimental comparison of convenience samples **Sample:** 122 Latino and black families with children 2-5.9 years presenting at two urban intervention (n=49) and comparison (n=73) clinics. | **Findings:** None reported  **SDoH:** Increased change in parent-child literacy-building activities and child language development.  **Provider:** None reported **Health:** None reported **Cost/Utilization:** None reported |
| 37 | Seligman et al., 2015^80^ | Food Insecurity | Food for Families identifies food insecure families during clinic visit and connects them with food resources (e.g., SNAP, WIC, and  food pantries). | Primary care | **Study design:** Pre/post comparison  **Sample:** 687 adult diabetic food pantry clients | **Findings:** Majority of clients preferred diabetic food box to regular food pantry options and consumed most or all of its contents. All implementation sites adopted intervention components.  **SDoH:** None reported  **Provider:** None reported  **Health:** Improvement in glycemic control; fruit and vegetable intake; diabetes self-efficacy and medication adherence.  **Cost/Utilization:** None reported |
| 38 | Coker et al.,  2012^81^ | IPV | Clinic-based IPV  advocate provides  needs assessment,  safety planning,  education, and  referrals to additional  resources. | Rural primary care | **Study design:** Quasi-  experimental longitudinal  cohort followed for 24  months.  **Sample:** 231 women with  Increased IPV screen (138  intervention clinic, 93  comparison clinics). | **Findings:** None reported  **SDoH:** Reduction in IPV scores.  **Provider:** None reported  **Health:** Reduction in depressive symptoms.  **Cost/Utilization:** None reported |
| 39 | Sanders et al, 2000^82^ | Education / Literacy | Reach Out and Read (ROR), families with babies and young children receive books and literacy guidance at well- child visits. | Pediatric primary care | **Study design:** Cross- sectional comparison of convenience sample  **Sample:** 122 predominantly Hispanic immigrant parents of children aged 2 months to 5 years who received books (n=56) or had not (n=66) from the pediatrician. | **Findings:** None reported  **SDoH:** Increased change in parent-child literacy-building activities.  **Provider:** None reported  **Health:** None reported  **Cost/Utilization:** None reported |
| 40 | Silverstein et al, 2002^83^ | Education / Literacy | Reach Out and Read (ROR), families with babies and young children receive books and literacy guidance at well- child visits. | Children and teen clinic | **Study design:** Quality Improvement pre-/ post- comparison of two cross- sectional cohorts **Sample:** 180 parents of children 6 months to 5.5 years were assessed pre- intervention (n=95) and post-intervention (n=85) implementation. | **Findings:** None reported  **SDoH:** Increased change in parent-child home reading activities.  **Provider:** None reported **Health:** None reported **Cost/Utilization:** None reported |
| 41 | Krasnoff and Mascati, 2002^84^ | IPV | ED-based screening and linkage to community-based IPV case management | Urgent care | **Study design:** Observational case study **Sample:** 528 women who screened positive for IPV in urgent care visit. | **Findings:** Increased change in use of IPV advocate; Increased change in use of community case management follow-up services.  **SDoH:** Reduced participant belief that they were at-risk for IPV.  **Provider:** None reported  **Health:** None reported  **Cost/Utilization:** None reported |
| 42 | Short et al.,  2002^85^ | IPV | WomanKind, hospital-based program designed to train providers and  personnel to identify domestic violence.  Patient advocates  provide referrals to  community agencies. | Hospital | **Study design:** Quasi- experimental longitudinal analysis of  data collected in 1996 and 1997.  **Sample:** 327 staff from  three interventions (n=200)  and two comparisons  hospitals (n=127). | **Findings:** Increased change in number of referrals for IPV.  **SDoH:** Reduced participant belief that they were at-risk for IPV.  **Provider:** Increased change in provider knowledge, attitudes and beliefs about IPV.  **Health:** None reported  **Cost/Utilization:** None reported |
| 43 | McCaw et al., 2001^86^ | IPV | Family Violence Prevention Project, a systems model approach using tools for effective referral, evaluation, and reporting of domestic violence; (2) materials for distribution to female patients; (3) training for social service and mental health clinicians to provide domestic violence evaluation; and  (4) strong links to the community. Screening  provided by frontline clinicians. | Primary and urgent care | **Study design:** Pre/post comparison of two random samples drawn in May 1998 and May 1999  **Sample:** 397 female health plan members who had routine visits in internal medicine or OB/GYN (190 pre-  intervention, 207 post- intervention). | **Findings:** Increased change in numbers of patients receiving social screening and referrals to domestic violence specialist; Increased change in-patient satisfaction with health plan’s efforts to address IPV.  **SDoH:** None reported **Provider:** None reported **Health:** None reported  **Cost/Utilization:** None reported |
| 44 | Gillum et al., 2009^87^ | IPV | Clinic-based screening and CHW counseling intervention aimed at engaging women in safety-promoting behaviors. | Primary care clinic for uninsured women | **Study design:** Randomized controlled pilot study  **Sample:** 41 women who screened positively for IPV were randomized into CHW intervention (n=20) or control (n=21). | **Findings:** None reported  **SDoH:** Increased change in safety-promoting behaviors.  **Provider:** None reported  **Health:** None reported  **Cost/Utilization:** None reported |

| 45 | Teufel et al., 2009^88^ | Legal needs | Medical-Legal Partnership of Southern Illinois (MLPSI), Medical- Legal Partnership targeting underserved people living in  southern Illinois. | Rural hospital system | **Study design:** Secondary analysis of 5 years of documentation collected between 2002-2006  **Sample:** 428 patients referred to MLPSI. | **Process:** Increased change in number of identified legal needs.  **SDoH:** Increased change in number of resolved legal issues.  **Provider:** None reported  **Health:** None reported  **Cost/Utilization:** Increased recovery of healthcare dollars and return on investment. |
| --- | --- | --- | --- | --- | --- | --- |
| 46 | Ulbrich and  Stockdale, 2002^89^ | IPV | RADAR, IPV  intervention focused  on routine IPV  screening by  providers, safety  assessments, and  referrals to  community-based  IPV advocacy  partners. | Rural family  planning clinics | **Study design:** Pre/post  survey  **Sample:** 16 clinical staff  from a network of rural  family planning clinics. | **Process:** Increased change in clinicians conducting routine  screening for IPV.  **SDoH:** None reported  **Provider:** None reported  **Health:** None reported  **Cost/Utilization:** None reported |
| 47 | Zachary et al.,  2002^90^ | IPV | IPV provider  training, on-site IPV  coordinator and  public health  campaign. | Urban family  practice | **Study design:** Pre/post  survey and focus group  **Sample:** 27 healthcare  providers serving pregnant  women in an urban family  practice. | **Process:** Providers highlighted the importance of an  easily accessible IPV coordinator, but did not find  protocol materials useful.  **SDoH:** None reported.  **Provider:** Increased change in provider confidence and self-  efficacy in the care of pregnant women experiencing IPV. No overall change in IPV attitudes or knowledge.  **Health:** None reported  **Cost/Utilization:** None reported |
| 48 | Smith et al., 2017^91^ | Food Insecurity | Student-run Free Clinic (SRFC) Food insecurity screening and referral program. | Student-run free clinics | **Study design:** Cross- sectional survey **Sample:** 430 adult patients presenting for  care in three SRFCs from January through July 2015. | **Process:** Increased identification of food insecurity, referrals, and use of clinic, community, and public assistance food programs. Program implemented as intended with no known inconsistencies.  **SDoH:** None reported **Provider:** None reported **Health:** None reported  **Cost/Utilization:** None reported |
| 49 | Pettignano et al., 2011^92^ | Legal needs | The Health Law Partnership (HeLP), Medical-Legal Partnership based in pediatric care. | Pediatric primary care | **Study design:** Retrospective cohort descriptive analysis of data collected between April 2004 and September 2010  **Sample:** 71 parents or guardians of 76 children with a diagnosis of sickle cell disease seen by lawyers of HeLP. | **Process:** Increased change in identifying and closing legal cases. **SDoH:** Increased change in benefits enrollment (e.g., disability benefits, employment, education, custody, housing repairs).  **Provider:** None reported  **Health:** None reported  **Cost/Utilization:** None reported |
| 50 | Nguyen et al.,  2016^93^ | Multiple SDoH (housing, transportation, food, clothing, dental and prescription services, employment, or family social services) | Health Connectors Program, volunteers provide social needs screening in waiting room and connect  families to resources. | Federally Qualified Health Center (FQHC) primary care | **Study design:** Pre/post surveys  **Sample:** 28 older diabetic adults. | **Process:** Increased identification of social needs; Half of patients reported contacting referrals provided to them.  **SDoH:** None reported  **Provider:** None reported  **Health:** No change in diabetes self-care or self-efficacy.  **Cost/Utilization:** None reported |
| 51 | Onyekere et al., 2016^94^ | Multiple SDoH( Education, employment, poverty, housing) | Medical Student Advocate, volunteer osteopathic medical students integrated into care team to assess and address patients’ nonmedical social needs. | Patient Centered Medical Home (PCMH) | **Study design:** Descriptive analysis of program data collected between August 2013 to August 2015; qualitative analysis of reflection student reflection sessions.  **Sample:** 31 medical student advocates | **Process:** Increased identification of nonmedical needs and connections of patients to community-based resources.  **SDoH:** None reported  **Provider:** Medical student advocates reported that program increased their empathy toward patients, understanding of social determinants of health, confidence entering third-year clerkships and also continued to address social needs during third-year clerkships.  **Health:** None reported  **Cost/Utilization:** None reported |
| 52 | Pettignano et al., 2012^95^ | Legal needs | The Health Law Partnership (HeLP), Medical-Legal Partnership based in pediatric care. | Pediatric primary care | **Study design:** Retrospective cohort descriptive analysis of data collected on cases served by HeLP between April 2006 through June 2010  **Sample:** Cases involving attaining or retaining Medicaid coverage and cases involving Medicaid denial of services. | **Process:** None reported  **SDoH:** None reported  **Provider:** Increased change in physician satisfaction.  **Health:** None reported  **Cost/Utilization:** Increased change in receipt of previously unreimbursed Medicaid payments. Providers also reported qualitatively that HeLP decreased emergency department visits, decreased readmissions, and helped decrease length of stay. |
| 53 | Beck et al., 2012^96^ | Legal needs | Child HeLP, Medical-Legal Partnership targeting families with legal needs presenting in pediatric primary  care. | Pediatric primary care | **Study design:** Descriptive case study analysis **Sample:** Families with children living in substandard housing cluster identified. | **Process:** Increased change in identification of environmental risks in substandard housing cluster.  **SDoH:** Increased change in resolved environmental risks.  **Provider:** None reported  **Health:** None reported **Cost/Utilization:** None reported |
| 54 | Klein et al.,  2013^97^ | Legal needs | Child HeLP, Medical-Legal Partnership targeting families with legal needs presenting in pediatric primary.  care. | Pediatric primary care | **Study design:** Descriptive analysis of Child HeLP cases between January 2009 and December 2011. **Sample:** Referrals made to MLP (n=1,808) from three pediatric primary care centers serving high- risk patients. | **Process:** Increased change in number of legal referrals from all levels of providers.  **SDoH:** Increased change in number of resolved legal issues.  Increased change in recovered back benefits.  **Provider:** None reported  **Health:** None reported **Cost/Utilization:** None reported |
| 55 | Zheng et al., 2018^98^ | Poverty | Psychosocial Assessment Tool 2.0 (PAT): a two-item screen for household material hardship (HMH) | In-hospital | **Study design:** Retrospective cohort from 2013 to 2017,  **Sample:** Referrals made to pediatric oncology center | **Process :** Increased identification of household material hardship (HMH)- Housing (24%), utilities (20%) and transportation (20%)  **SDoH:** None reported.  **Provider:** None reported  **Health:** Not reported.  **Cost/Utilization:** Not reported |
| 56 | Kurani et al.,2020^99^ | Multiple SDOH (income, housing, employment, and education) | Area deprivation index (ADI) | Primary care | **Study design:** Cross-sectional study of adults receiving primary care using electronic health records between July 1, 2016, and June 30, 2017.  **Sample:** breast, cervical, and colorectal cancer patients from 75 primary care practices in 3 US Midwest states (Minnesota, Iowa, and Wisconsin) | **Process:** Patients in areas of greater deprivation and rurality had lower rates of cancer screening, signaling the need for effective intervention strategies to enhance access to screening in highest-risk populations.  **SDoH:** None reported.  **Provider:** None reported  **Health:** None reported  **Cost/Utilization:** None reported |
| 57 | Berkowitz et al., 2021^100^ | Multiple SDOH(financial resource, transportation, stress, depression, intimate partner violence, social connections, physical activity, and alcohol consumption) | Epic-compatible paper questionnaire | Ambulatory clinic | **Study design:** Descriptive analysis  **Sample:** Patients receiving Medicare wellness, adult annual, or new patient visits during a five-week period (February-March 2020) | **Process:** Increased identification of patients with at least 1 social need, most commonly stress followed by need for physical activity.  **SDoH:** None reported.  **Provider:** Average length of visit was 39.8 min, which was 1.7 min longer than that in 2019. Visit lengths were longer among patients 65+ (43.4 min) and patients having public insurance (43.6 min).  **Health :** None reported  **Cost/utilization:** None reported |
| 58 | Stenson et al., 2018^101^ | Multiple SDOH (Behavioral health services, education, employment, housing, utilities, Social support and housing) | questionnaire | Outpatient settings | **Study design:** Descriptive analysis  **Sample:** Participants were aged 18 years or older, spoke English, had been released for a least 1 month, and had at least 1 child younger than 4 years at the time of release. | **Process:** Increased identification of mothers who had been released from an urban jail about eight domains of community reentry emerged through analysis.  **SDoH :** behavioral health services, education, employment, housing, material resources, medical care, relationships with children, and social support.  **Provider :None reported**  **Health :None reported**  **Cost/utilization: None reported** |
| 59 | Buitron et al.,2019^102^ | Multiple SDOH (Housing, Food, Paying for medicines, Transportation, utilities, childcare, Care for elderly, job search, and education) | BMC THRIVE (Modified WE CARE) screening model | Primary care | **Study design:** Descriptive analysis  **Sample:** Patients who were screened to be positive and requested resources for social needs | **Process:** Successful implementation of a systematic clinical strategy in primary care using EHR workflows.  **SDoH:** Connected the patients to necessary resources for their SDOH needs.  **Provider :None reported**  **Health :None reported**  **Cost/utilization: None reported** |
| 60 | Costitch et al., 2019^103^ | Multiple SDOH( caregiver distress, education, isolation, food, and housing) | CHW program for caregivers of children with special health care needs | Ambulatory Care Network – pediatric practice | **Study design:** Retrospective pre-post analysis  **Sample:** Patients who have completed an Ambulatory Care Network program | **Process:** Caregivers reported high levels of distress, low educational attainment, linguistic isolation, positive depression screens, and food and housing issues at baseline. Successful implementation of community health worker (CHW) intervention  **SDoH:** Significant improvement in caregiver distress scores and understanding of child’s pathology.  **Provider :None reported**  **Health :None reported**  **Cost/utilization: None reported** |
| 61 | Cusak et al., 2019^104^ | Housing instability | EHR compatible screening of SDOH | Outpatient settings | **Study design :** Retrospective observational study  **Sample:** The cohort included Veterans who had responded to the HSCR in the 90 days prior to presenting for SSVF services between October 2012 and 2015 (N = 134). | **Process:** Increased triage assistance  **SDoH:** None  **Provider:** Improved connection with community resources  **Health :**None  **Cost/utilization:** None |
| 62 | Power-Hays et al., 2020 ^105^ | Multiple SDOH (housing, food, employment, childcare, education, utilities, transportation to the hospital, and ability to pay for medications) | Screening for SDoH using WE CARE model. | Pediatric hematology clinic | **Study design:** Prospective, quality-improvement study  **Sample:** SDoH in a busy subspeciality clinic from august 2017 to November 2018. | **Process:** a paper screener followed by a referral to local community organizations for the specific needs 66% patients had at least 1 social need.  **SDoH:** None reported  **Provider:** Increased connection with community resources  **Health :None reported**  **Cost/utilization: None reported** |
| 63 | Albright et al., 2021^106^ | Multiple SDoH (Education, housing stability, and employment status) | SDOH screening tool for level of education, housing stability, and employment status | Primary care | **Study design:** This survey-based study focused on a patient population located in rural west Alabama surveyed for a screening, brief intervention, and referral to treatment program.  **Sample:** Patients with self-reported use of Opioids | **Process:** Association of opioid use and SDOH parameters  **SDoH:** Adjusted incidence rate ratios (IRRs) for the relationship between social determinants of health and opioid use frequency (in days/month)  **Provider: None reported**  **Health : None reported**  **Cost/utilization: None reported** |
| 64 | Rogers et al., 2022^107^ | Multiple SDoH(housing instability, food insecurity, transportation problems, utilities, and interpersonal safety) | Centers for Medicare & Medicaid Services (CMS) Center for Medicare and Medicaid Innovation's (CMMI) Accountable Health Communities (AHC) Model. | Inpatient and outpatient | **Study design:** Prospective observational study.  **Sample:** Medicare and Medicaid beneficiaries | **Process:** Increased integration of SDOH screening with EHR  **SDoH:** housing instability, food insecurity, transportation problems, utilities, and interpersonal safety  **Provider:** All Medicare and Medicaid beneficiaries who screen positive for at least one SDOH receive a tailored Community resource specialist upon discharge or checkout  **Health :None reported**  **Cost/utilization: None reported** |
| 65 | Crusan et al., 2023^108^ | Food insecurity, Transport and Money | Community survey | Outpatient clinic | **Study design :** Qualitative study.  **Sample:** Hispanic and Latino community in the community-based clinic | **Process:** The food insecurity questionnaires showed high/marginal (40%), low (53.3%), and very low (6.7%) food security.  **SDoH:** None reported.  **Provider:** None  **Health** :None  **Cost/utilization:** None |
| 66 | Javed, Zulqarnain et al, 2023^27^ | Multiple SDOH (employment status, family income, housing, psychological distress, education, insurance status, and lack of transportation) | National Health Interview Survey linked to National Death index | Secondary analysis | **Study design:** Cross sectional study of participants from 2006 to 2018.  **Sample:** Non-Hispanic black and non-Hispanic White adults**.** | **Process :**Increased racial inequities in all-cause mortality and cardiovascular disease mortality associated with high burden of SDOH.  **SDoH:** None reported.  **Provider:** None reported  **Health :** None reported  **Cost/utilization:** None reported. |
| 67 | Javed, Zulqarnain et al., 2022^109^ | Multiple SDOH () economic stability; 2) neighborhood, physical environment, and social cohesion; 3) community and social context; 4) food insecurity; 5) education; and 6) health care system) | National Health Interview Survey | Secondary analysis | **Study design:** Cross sectional study of participants from 2013to 2017  **Sample:** Data for 161,795 adults aged ≥18 years from the 2013 to 2017 National Health Interview Survey were used. | **Process :** There was a graded increase in obesity prevalence with increasing SDOH burden.  **SDoH:** None reported.  **Provider:** None reported  **Health :** None reported  **Cost/utilization:** None reported. |
| 68 | Abar et al, 2016^110^ | Lack of transportation | Survey | Emergency Department | **Study design:** Cross sectional study  **Sample:** A total of 636 participants were enrolled. The percentage of participants with mild or greater depression was 42%. | **Process:** The majority of patients reported experiencing some barriers to care  **SDoH:** None reported.  **Provider:** None reported  **Health :** None reported  **Cost/utilization:** None reported. |
| 69 | Sood et al., 2021^111^ | Healthcare access | Survey | Outpatient settings | **Study design:** Randomized controlled trial  **Sample:** Uninsured NYC residents aged 19 years or older with household incomes less than or equal to 200% of the federal poverty level, according to the Department of Health and Human Services, were eligible if they were ineligible for insurance. | **Process:** Increase in access to healthcare and doctor visits after intervention.  **SDoH:** None reported.  **Provider:** None reported  **Health :** None reported  **Cost/utilization:** None reported. |
| 70 | Ireson et al.,^112^ | Socioeconomic risk screening | Interviews | Inpatient settings | **Study design: cross sectional study**  **Sample:** This study included 179 telemedicine encounters in a pediatric primary care hospital. | **Process:** The screening tool was completed in 63% of encounters and was positive in 5% of encounters.  **SDoH:** Of those who identified socioeconomic risks, 90% received a referral/intervention (social work consultation, food pantry, etc.).  **Provider:** None reported  **Health :** None reported  **Cost/utilization:** None reported. |
| 71 | Baidal et al., 2023^113^ | Food insecurity | Accountable Health Communities health-related needs screening tool, which includes the 2-item Hunger Vital Signs™ | Inpatient settings | **Study design:** Prospective observational study  **Sample:** Patients age <6 years with a routine care visit, the completion of food insecurity screening questions in the pre-intervention period, and at least one anthropometric measurement in the pre-intervention (May 22, 2014–July 16, 2019)and post-intervention (July 29, 2019–March 20, 2020) periods were included. | **Process:** Improvement in BMI of children post 6 months after intervention  **SDoH:** None reported.  **Provider:** None reported  **Health :** None reported  **Cost/utilization:** None reported. |
| 72 | Inoue et al., 2022^114^ | Social isolation in racial minority groups | Self- declaration in the SPRINT clinical trial. | Outpatient settings | **Study design:** Post hoc analysis of SPRINT trial  **Sample:**  A multicenter study of 9342 individuals with increased risk for cardiovascular disease and free of diabetes, enrolled at 102 clinical sites in the United States between November 2010 and March 2013. | **Process:** None  **SDoH:** None reported.  **Provider:** None reported  **Health :** After 3 years of intensive blood pressure treatment, Black individuals living alone had significantly lower cardiovascular outcomes  **Cost/utilization:** None reported. |
| 73 | Martin et al.,2023 ^115^ | Multiple SDOH (Food insecurity, employment, housing) | Quantitative surveys and Qualitative interviews. | Outpatient settings | **Study Design:** Cross sectional study  Sample : Latinx adults ≥ 18 years old | **Process:** Primary barriers for the Latinx community during the COVID-19 pandemic included insecurities in food, jobs, housing, and immigration.  **SDoH:** None reported.  **Provider:** None reported  **Health :** None reported  **Cost/utilization:** None reported. |
| 74 | Jamerson et al., 2017^116^ | Socioeconomic status | Surveys in a school-based intervention designed to reduce the risk of obesity and CVD in children through the promotion of healthy eating and physical activity | Outpatient settings | **Study design:** Cross sectional study  Sample: A total of 3813 sixth-grade students comprised the survey sample, and 2297 sixth-grade students comprised the screening sample. | **Process:** At baseline, blacks had a higher percentage of overweight/obese students (43% vs 34%; P < .0001) and demonstrated poorer health habits than non-blacks; however, non-blacks had poorer lipid profiles.  **SDoH:** None reported.  **Provider:** None reported  **Health :** At follow-up (post- intervention), both groups demonstrated significant improvements in physiological measures and health behaviors.  **Cost/utilization:** None reported. |
| 75 | Wetherhill et al., 2018^117^ | Access to healthy foods | The Food Choice Values (FCV) Questionnaire uses 25 items to assess eight FCV subscales related to buying and eating food. | Outpatient settings | **Study design:** Cross sectional  **sample:** 83 American Indian patrons shopping at tribally owned convenience stores ≥3 times per week. | **Process:** cluster analysis identified four groups, or segments, each with distinct patterns of FCV endorsement: limited endorsement of any FCVs (23.3%); safety and sensory appeal (32.9%); health/weight control (17.8%); and broad endorsement of FCVs (26.0%).  **SDoH:** None reported.  **Provider:** None reported  **Health :** None reported  **Cost/utilization:** None reported. |
| 76 | Gerber et al., 2020^118^ | Multiple SDOH (Food insecurity, homelessness,employment) | Surveys | Emergency department | **Study design:** Cross sectional study  **Sample:** Study surveyed a random sample of 2312 public hospital ED patients. Surveys included validated single-item screeners for unhealthy alcohol and any drug use and questions on self-reported past-year material needs. | **Process:** Prevalence of health-related material needs-HRMNs for all patients-including food insecurity (50.8%), inability to meet essential expenses (40.8%), cost barriers to medical care (24.6%), employment issues (23.8%), and homelessness (21.4%)-was high.  **SDoH:** None reported.  **Provider:** None reported  **Health :** None reported  **Cost/utilization:** None reported. |
| 77 | Steinman et al.,2021^119^ | Social isolation | Brief validated social connectedness scales: Duke Social Support Index 10-item (DSSI-10), PROMIS-Social Isolation (6-item), UCLA-Loneliness (3-item); | Community-based social service organizations (N = 16) in five U.S. states | **Study design:** Multisite, pre-post single-group evaluation  **Sample:** Multisite, pre-post single-group evaluation | **Findings:** participants significantly increased social interactions and satisfaction with social support and reduced perceived isolation; and loneliness.  **SDoH:** None reported  **Provider:** None reported  **Health:** Increased social connectedness was associated with reduced depression. Improvements in social connectedness (except social interactions) persisted during early COVID-19.  **Cost/utilization:** None reported. |
| 78 | Ijadi-Maghsoodi et .,2019^120^ | Homelessness | Semi-structured interviews | Outpatient settings | **Study design:** Cross sectional study  **Sample:** a total of 41 in-depth semistructured interviews with parents with a history of SUDs and homelessness (n = 16) and housing support staff (n = 25) from May 2017 until January 2018. | **Findings: A** high burden of trauma and guilt exists among parents, and a fear of substance use disorder disclosure.  **SDoH:** None reported.  **Provider:** None reported  **Health:** We found challenges with family processes important for SUD prevention, including communication, discussion of substance use, and family and youth goal setting.  **Cost/utilization:** None reported. |

Findings: represents the outcomes of health related social needs screening. SDoH represents change in SDoH variables post intervention; Health: Changes in the health outcomes post intervention. Cost/Utilization: Cost changes post intervention. Provider: Changes or effects at healthcare provider level observed as a result of SDoH screening.

Studies were assigned ratings reflecting methodologic quality based on the Grading Recommendations Assessment Development and Evaluation (GRADE) approach to grading evidence quality (high, medium, low, very low).^8,121^

**Appendix Table 2 . Psychometric Evidence for Social Risk Screening Tools^122^**

|  | Internal consistency | Convergent Validity | Discriminant Validity | Known groups validity | Predictive validity | Concurrent validity | Structural validity | Responsiveness | Norms | Total |
| --- | --- | --- | --- | --- | --- | --- | --- | --- | --- | --- |
| WE CARE-BMC THRIVE | 0 | 0 | 0 | 0 | 0 | 0 | 0 | 0 | 2 | 2 |
| The Health Leads screening toolkit | 0 | 0 | 0 | 0 | 0 | 0 | 0 | 0 | 0 | 4 |
| PRAPARE tool | 0 | 4 | 0 | 0 | -1 | 0 | 0 | 0 | 2 | 5 |
| The Accountable Health Communities-HRSN tool | 0 | 0 | 0 | 0 | 0 | 0 | 0 | 0 | 0 | 0 |
| CDC Environmental justice index (EJI) | NA | NA | NA | NA | NA | NA | NA | NA | NA | NA |
| EPIC SDOH tool | NA | NA | NA | NA | NA | NA | NA | NA | NA | NA |

Note: Scores within each category range from ‒1 (poor) to 4 (excellent). The minimum total score is ‒9, the maximum total score is 36. NA- Not available

**Appendix Table 3. Pragmatic Evidence for Social Risk Screening Tools^122^**

|  | **Cost** | **Accessible language** | **Ease of Training** | **Ease of Interpretation** | **Brief** | **Total Score** |
| --- | --- | --- | --- | --- | --- | --- |
| **WE CARE-BMC THRIVE** | 4 | 4 | 4 | 1 | 4 | 17 |
| **The Health Leads screening toolkit** | 3 | 4 | 2 | 1 | 4 | 13 |
| **PRAPARE tool** | 4 | 4 | 2 | 1 | 3 | 14 |
| **The Accountable Health Communities-HRSN tool** | 4 | 3 | 0 | 0 | 3 | 10 |
| **CDC Environmental justice index (EJI)** | NA | NA | NA | NA | NA | NA |
| **EPIC SDOH tool** | NA | NA | NA | NA | NA | NA |

Note: Scores within each category range from ‒1(poor) to 4 (excellent). The minimum total score is‒5, the maximum total score is 20. NA- Not available

The different categories of outcomes provide insights into the impacts of social determinants of health (SDOH) interventions at various stages: Direct and intermediate process measures track whether the intervention was implemented as intended. This includes things like screening rates, referrals provided, and patient satisfaction.^8^ Social determinant of health outcomes measure the intervention's direct impacts on social and economic factors like food security, housing, and employment.^8^ Immediate, intermediate, and longer-term health impacts assess changes in health status, quality of life, disease control, etc. These are key outcomes but may take time to manifest. Healthcare utilization/cost impacts analyze effects on ED visits, hospitalizations, costs, etc. ^8^These are critical to understanding financial implications. Provider costs and outcomes evaluate impacts on providers, like satisfaction, productivity, and confidence addressing SDOH.^8^ This helps sustain programs. Tracking outcomes across this framework provides a comprehensive picture of an SDOH intervention's clinical and financial effects.^8^ Process measures ensure it was implemented properly before assessing downstream impacts.^8^ Strong SDOH and health outcomes build the case for population health benefits. Utilization/cost impacts make the business case to healthcare administrators. ^8^ Provider outcomes help gain clinician buy-in. Evaluating across multiple outcome types paints a complete picture of the intervention's effects.^8^

**Appendix Table 4 . References for the SDOH domain screening survey of the proposed tool**

| **Tool SDOH Domain** | **Item #** | **Question** | **Reference** |
| --- | --- | --- | --- |
| Minority Status | 1,2 | What is your race?  What is your ancestry or ethnic origin? | United States Census Bureau. The American Community Survey. American Community Survey. Published 2022. Accessed May 30, 2023. www2.census.gov/programs-surveys/acs/methodology/questionnaires/2022/quest22.pdf |
| Socio-economic factors | 3,4,5,10 | What was your total household income during the PAST 12 MONTHS?  What is your highest degree or level of school you have COMPLETED?  Are you currently employed?  If “yes” to the above, please answer item 5a) below. If “no” to the above, please skip to item    5a. a) Full-time? b) Part-time c) Variable  5b). When did you last work, even for a few days?  Are you CURRENTLY covered by any of the following types of health insurance or health coverage plans?    10a.Insurance through a current or former employer or union (of this person or another family member)?  10b. Insurance purchased directly from an insurance company (by this person or another family member)?  10c. Medicare, for people 65 and older, or people with certain disabilities?  10d. Medicaid, Medical Assistance, or any kind of government-assistance plan for those with low incomes or a disability?  10e. TRICARE or other military health care?  10f. VA (enrolled for VA health care)?  10g. Indian Health Service?  10h. Any other type of health insurance or health coverage plan1 – Specify _________________ | United States Census Bureau. The American Community Survey. American Community Survey. Published 2022. Accessed May 30, 2023.  www2.census.gov/programs-surveys/acs/methodology/questionnaires/2022/quest22.pdf  National Center for Health Statistics. National health Interview Survey.  <https://ftp.cdc.gov/pub/health_Statistics/nchs/Survey_Questionnaires/NHIS/2018/english/qadult.pdf> |
| Financial resource strain | 6c | What is the monthly mortgage, loan or rent for this house, apartment, or mobile home? | United States Census Bureau. The American Community Survey. American Community Survey. Published 2022. Accessed May 30, 2023. www2.census.gov/programs-surveys/acs/methodology/questionnaires/2022/quest22.pdf |
| Household Type | 6,7 | 6a.What is your housing situation today?  6b.Is this house, apartment, or mobile home?  7.Which best describes your building? | United States Census Bureau. The American Community Survey. American Community Survey. Published 2022. Accessed May 30, 2023. www2.census.gov/programs-surveys/acs/methodology/questionnaires/2022/quest22.pdf |
| Household characteristics | 8,14 | 8a. How many people are living or staying at your home address?  8b. How many separate bedrooms are in this house, apartment or mobile home?  14a. What is your marital status?  14b. Are you a single parent? (Male/female householder, no spouse or partner present, with own children under 18 years) | United States Census Bureau. The American Community Survey. American Community Survey. Published 2022. Accessed May 30, 2023. www2.census.gov/programs-surveys/acs/methodology/questionnaires/2022/quest22.pdf |
| Environment Burden | 9 | How long have you been at your current residence?  What is the zip code of your longest-lived residence? | Ompad, D. C., Galea, S., Caiaffa, W. T., & Vlahov, D. (2007). Social determinants of the health of urban populations: methodologic considerations. Journal of urban health : bulletin of the New York Academy of Medicine, 84(3 Suppl), i42–i53. <https://doi.org/10.1007/s11524-007-9168-4> |
| Access to Phone and Internet | 11 | At this house, apartment, or mobile home –do you or any member of this household have access to the Internet | United States Census Bureau. The American Community Survey. American Community Survey. Published 2022. Accessed May 30, 2023. www2.census.gov/programs-surveys/acs/methodology/questionnaires/2022/quest22.pdf |
| Disability | 12 | 12a. Are you deaf or do you have serious difficulty hearing?  12b. Are you blind or do you have serious difficulty seeing, even when wearing glasses?  12c. Because of a physical, mental, or emotional condition, do you have serious difficulty concentrating, remembering, or making decisions?  12d. Do you have serious difficulty walking or climbing stairs?  12e. Do you have difficulty dressing or bathing?  12f. Because of a physical, mental, or emotional condition, do you have difficulty doing errands alone such as visiting a doctor's office or shopping? | 2021 BRFSS Questionnaire. Behavioral Risk Factor Surveillance System. <https://www.cdc.gov/brfss/questionnaires/pdf-ques/2021-BRFSS-Questionnaire-1-19-2022-508.pdf>  Centers for Medicare & Medicaid Services. The Accountable Health Communities Health-Related Social Needs Screening Tool. Centers for Medicare & Medicaid Services. Published 2023. Accessed May 30, 2023. [https://innovation.cms.gov/files/worksheets/ahcm-screeningtool.pdf](about:blank) |
| Language | 13 | How well do you speak English? | United States Census Bureau. The American Community Survey. American Community Survey. Published 2022. Accessed May 30, 2023. www2.census.gov/programs-surveys/acs/methodology/questionnaires/2022/quest22.pdf |
| Transportation | 15. | 15a. How did you usually get to work LAST WEEK? Mark (X) ONE box for the method of transportation used for most of the distance?  15b. In the past 12 months, has lack of reliable transportation kept you from medical appointments, meetings, work, or from getting things needed for daily living? | United States Census Bureau. The American Community Survey. American Community Survey. Published 2022. Accessed May 30, 2023. www2.census.gov/programs-surveys/acs/methodology/questionnaires/2022/quest22.pdf |
| Access to food, utilities, and healthcare | 16 | In the past year, have you or any family members you live with been unable to get any of the following when it was really needed? Check all that apply.  -Food  -Healthy food (fresh fruits and vegetables)  -Utilities  -Medicines or Healthcare (Medical, Dental, Mental Health, Vision)  -Phone  -I choose not to answer this  If you responded “yes” to any of the above, please answer the following questions:  a) DURING THE PAST 12 MONTHS, please indicate if you or anyone in the family had problems paying or were unable to pay any medical bills?  b) DURING THE PAST 12 MONTHS, has medical care been delayed because of worry about the cost?  c) How worried are you right now about not being able to pay your rent, mortgage, or other housing costs?  d) How worried are you right now about not being able to pay your normal monthly bills? | National Association of Community Health Centers. Protocol for Responding to and Assessing Patient Assets, Risks, and Experiences. National Association of Community Health Centers. Published 2020. Accessed May 30, 2023. [https://prapare.org/](about:blank)  National Center for Health Statistics. National health Interview Survey.  https://ftp.cdc.gov/pub/health_Statistics/nchs/Survey_Questionnaires/NHIS/2018/english/qadult.pdf |
| Social Connection | 17 | How often do you see or talk to people that you care about and feel close to? (For  example: talking to friends on the phone, visiting friends or family, going to church or club meetings | National Association of Community Health Centers. Protocol for Responding to and Assessing Patient Assets, Risks, and Experiences. National Association of Community Health Centers. Published 2020. Accessed May 30, 2023. [https://prapare.org/](about:blank) |
|  | 18 | How much do you agree or disagree with the following statements about your neighborhood? Would you say… There are people I can count on in this neighborhood? | National Center for Health Statistics. National health Interview Survey.  https://ftp.cdc.gov/pub/health_Statistics/nchs/Survey_Questionnaires/NHIS/2018/english/qadult.pdf |
| Stress | 19a and 19b | 19a.Over the last 2 weeks, how often have you been bothered by the following problems?  1. Little interest or pleasure in doing things.  2. Feeling down, depressed or hopeless  19b. How often does your partner:  A. Physically hurt you?  B. Insult you or talk down to you?  C. Threaten you with harm?  D. Scream or curse at you? | 19a. Kroenke, K., Spitzer, R. L., & Williams, J. B. (2003). The Patient Health Questionnaire-2: validity of a two-item depression screener. Medical care, 41(11), 1284–1292. <https://doi.org/10.1097/01.MLR.0000093487.78664.3C>  19b.Rabin, R. F., Jennings, J. M., Campbell, J. C., & Bair-Merritt, M. H. (2009). Intimate partner violence screening tools: a systematic review. American journal of preventive medicine, 36(5), 439–445.e4. <https://doi.org/10.1016/j.amepre.2009.01.024> |
| Health Behaviors | 20 | In the last 30 days, other than the activities you did for work, on average, how many days per week did you engage in moderate exercise (like walking fast, running, jogging, dancing, swimming, biking, or other similar activities)?  On average, how many minutes did you usually spend exercising at this level on one of those days? | Centers for Medicare & Medicaid Services. The Accountable Health Communities Health-Related Social Needs Screening Tool. Centers for Medicare & Medicaid Services. Published 2023. Accessed May 30, 2023. [https://innovation.cms.gov/files/worksheets/ahcm-screeningtool.pdf](about:blank) |
